# Mycemycins A–E, New Dibenzoxazepinones Isolated from Two Different Streptomycetes

**DOI:** 10.3390/md13106247

**Published:** 2015-09-30

**Authors:** Ning Liu, Fangying Song, Fei Shang, Ying Huang

**Affiliations:** 1 State Key Laboratory of Microbial Resources, Institute of Microbiology, Chinese Academy of Sciences, Beijing 100101, China; E-Mails: fussliu@126.com (N.L.); songfangying_@126.com (F.S.); 2 University of Chinese Academy of Sciences, Beijing 100049, China; 3 Analytical and Testing Center, Beijing University of Chemical Technology, Beijing 100029, China; E-Mail: sjynm220@126.com

**Keywords:** mycemycin, dibenzoxazepinone, deep-sea, *Streptomyces*, gene disruption

## Abstract

Five new dibenzoxazepinone derivatives, mycemycins A–E (**1**–**5**), were isolated from the ethanol extracts of mycelia of two different streptomycetes. **1** and **2** were isolated from an acidic red soil-derived strain, *Streptomyces* sp. FXJ1.235, and **3**–**5** from a *gntR* gene-disrupted deep-sea strain named *Streptomyces olivaceus* FXJ8.012Δ*1741*. The structures of mycemycins were elucidated by a combination of spectroscopic analyses, including 1D- and 2D-NMR techniques.

## 1. Introduction

Actinomycetes are a proven source of diverse natural products and account for more than 40% of the currently known microbial bioactive compounds, including antibiotics, enzyme inhibitors, immunoactive agents, *etc.* [1,2,3]. While the great majority of bioactive compound-producing actinomycetes are from terrestrial habitats, in recent years, marine actinomycetes have become an up-and-coming source of novel natural products [4,5]. At the same time, increasing genome sequencing data of actinomycetes exhibit far more putative secondary metabolic gene clusters than the “active” clusters and relevant natural compounds, which means that the vast majority of the secondary metabolic potential remains untapped [6], and reinforces the idea that reexamination of well-studied actinomycete strains is still meaningful [7]. To explore new products of these silent gene clusters, several approaches have been developed including culture medium optimization, strain improvement, manipulation of global or pathway-specific regulators, heterologous expression, ribosome engineering, co-cultivation, *etc.* [8].

**Figure 1:**
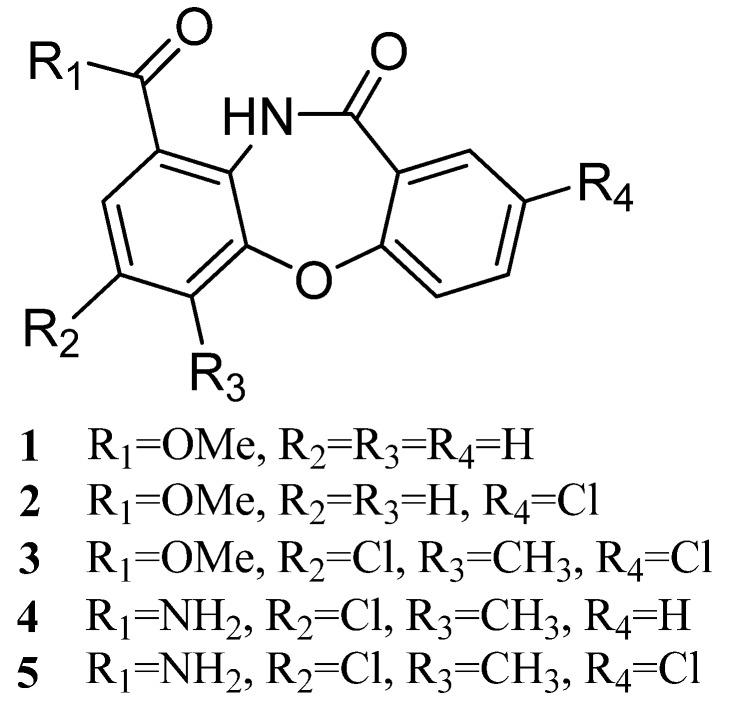
Structures of compounds **1**–**5**.

Recently, we reported tetroazolemycins and spoxazomicins from a deep-sea derived actinomycete, *Streptomyces olivaceus* strain FXJ8.012, which was isolated from a water sample collected from the south Indian Ocean [9]. A genome survey of this strain indicated that it contains a number of silent gene clusters that may be responsible for the biosynthesis of novel compounds, including halometabolites. By disruption of a putative transcriptional regulator gene *orf-1741*, we successfully activated the production of a cluster of novel natural products (**3**–**5**, Figure 1). Interestingly, these compounds exhibited similar UV spectra with another two compounds (**1**–**2**, Figure 1) that we have isolated from wild type *Streptomyces* sp. strain FXJ1.235, a terrestrial actinomycete derived from an acidic red soil sample collected from Jiangxi Province, China. Furthermore, all the compounds except **1** exhibited halogenated traits according to MS analysis. So we decided to study them together. Results demonstrated that they do possess high structural similarity. Herein we describe the isolation, structural elucidation and bioactivities of these novel compounds.

## 2. Results and Discussion

### 2.1. Genetic Manipulation of S. olivaceus FXJ8.012 and Isolation of Compounds **1***–***5**

In the genome of *S. olivaceus* FXJ8.012, *orf-1741* is flanked by putative secondary metabolic genes and encodes a 126-amino-acid peptide which was proposed as a member of GntR-family transcriptional regulator. GntR-like regulators play an important role in the control of actinomycete development and antibiotic biosynthesis [10,11]. Some members of the GntR family have previously been reported as repressors of secondary metabolite biosynthesis in actinomycetes. For example, MtdA represses nucleoside antibiotic A201A [12], SCO3269 represses actinorhodin and undecylprodigiosin [13], and ptnR1 represses platensimycin [14]. Thus, in order to activate the expression of related silent secondary metabolic gene clusters, we disrupted *orf-1741* by replacing it with a kanamycin-resistance gene (*neo*). The disruption resulted in a different HPLC-based metabolic profile of the mutant strain FXJ8.012Δ*1741* cultivated in modified R2 medium [15]. Consequently, three new halogenated dibenzoxazepinone derivatives (**3**–**5**) were detected from the mutant strain, while no new products were detected from the wild type strain (Supplementary Information Figure S1). Purification of the ethanol extracts of a 20 L fermentation culture of FXJ8.012Δ*1741* led to the isolation of mycemycins C–E (**3**–**5**).

In addition, purification of the ethanol extracts of a 10 L fermentation culture of *S.* sp. FXJ1.235 led to the isolation of mycemycins A and B (**1**–**2**), with much higher yields than **3** and **4** and better solubility than **5**. So we chose to elucidate the structures of compounds **1** and **2** first.

### 2.2. Structural Identification of Mycemycins A–E

Mycemycins A (**1**) was obtained as a white solid. Its molecular formula was established as C_15_H_11_NO_4_ according to the [M + H]^+^ peak at *m*/*z* 270.0776 (calcd for C_15_H_12_NO_4_, 270.0766) in the high resolution-electrospray ionization-mass (HR-ESI-MS) spectrum combined with the ^13^C-nuclear magnetic resonance (NMR) data, corresponding to 11 degrees of unsaturation.

The ^1^H NMR spectrum (Table 1 and Figure S2) showed seven aromatic protons, with four main doublets at δ 7.15, 7.81, 8.04 and 8.08 and three main triplets at δ 7.03, 7.46 and 7.48, and one methoxyl group at δ 4.06. Besides conventional coupling correlations, the aromatic “w” type coupling could also be found from protons at δ 7.48 and 8.04 with small *J* = 1.5 Hz. The ^13^C NMR spectrum (Table 1 and Figure S2) showed 15 carbon signals, and, combined with the DEPT and HSQC spectra (Figure S2), revealed one methoxyl group (δ 52.4), seven phenyl methine groups (δ 115.0, 117.7, 119.6, 124.8, 127.2, 127.5 and 134.3), and seven quaternary carbons (δ 110.0, 121.3, 139.4, 149.8, 159.4, and two possible ester/amido carbon signals at δ 164.4 and 165.6).

**Figure 2:**
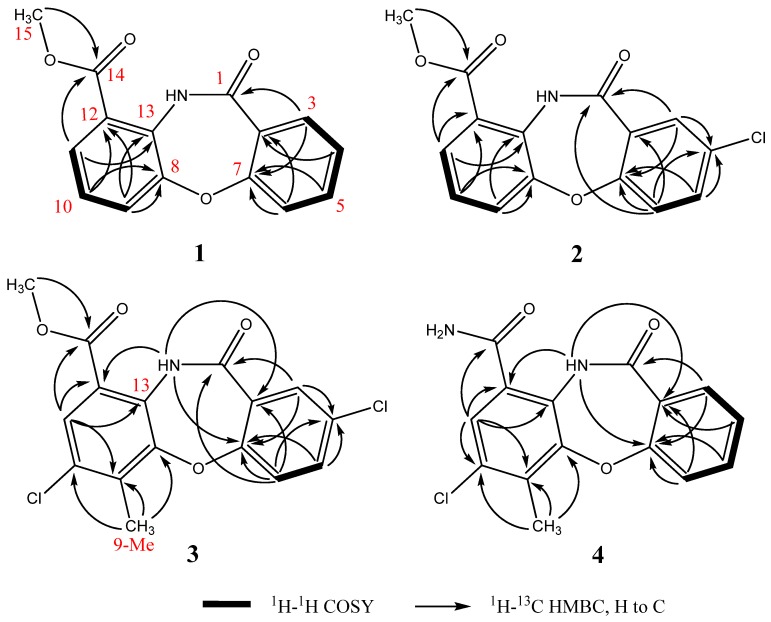
Key ^1^H-^1^H COSY and HMBC correlations of **1**–**4**.

Detailed analysis of 2D-NMR spectra (Figure 2 and Figure S2) revealed that **1** was comprised of two aromatic ring systems. The ^1^H-^1^H correlation spectroscopy (COSY) spectrum established the connections of H11(δ 8.08)-H10 (δ 7.46)-H9 (δ 8.04); the heteronuclear multiple bond correlation (HMBC) experiment revealed the key correlations from H11 and 15-Me to C14, H9 and H11 to C13, and from H9 and H10 to C12 and C8. According to the above data, the first aromatic ring from C8 to C15 was established. Furthermore, the chemical shifts of C15 (δ 52.4) and C8 (δ 149.8) indicated that both of the two carbons were connected to an oxygen atom. The COSY spectrum revealed the connection of H3 (δ 8.04)-H4 (δ 7.03)-H5 (δ 7.48)-H6 (δ 7.15); the HMBC correlations from H3 to C1 and from H4 and H6 to C2 suggested the connections of C1-C2-C3; the HMBC correlations from H3, H5 and H6 to C7 and the chemical shift of C7 (δ 159.4) revealed the connections of C2-C7-C6 where C7 was connected to an oxygen atom. Based on these data, the second aromatic ring from C1 to C7 was established. Moreover, according to the supposed molecular formula, the two ring systems should connect with each other though C7-O-C8 and C11-NH-C13.

**Table 1:** ^1^H- (500 MHz) and ^13^C-NMR (125 MHz) data of **1**–**4** in *CDCl_3_* (δ in ppm, *J* in Hz).

Position	1		2		3		4	
	δ_H_	δ_C_	δ_H_	δ_C_	δ_H_	δ_C_	δ_H_	δ_C_
1	―	164.4, C	―	163.1, C	―	163.3, C	―	163.9, C
2	―	110, C	―	111, C	―	110.8, C	―	109.8, C
3	8.04 (dd, 1.5, 8.0)	127.2, CH	8.01 (d, 2.5)	126.4, CH	8.00 (d, 2.5)	128.4, CH	8.10 (d, 7.5)	127.83, CH
4	7.03 (t, 7.5)	119.6, CH	―	124.5, C	―	124.5, C	7.10 (t, 7.5)	120.4, CH
5	7.48 (dt, 1.5, 7.8)	134.3, CH	7.42 (dd, 2.5, 8.5–9.0)	134.1, CH	7.43 (dd, 2.5, 8.5–9.0)	134.2, CH	7.53 (t, 7.5)	134.9, CH
6	7.15 (d, 8.5)	117.7, CH	7.10 (d, 9.0)	119.3, CH	7.10 (d, 9.0)	119.3, CH	7.15 (d, 8.0)	117.7, CH
7	―	159.4, C	―	157.9, C	―	157.9, C	―	158.4, C
8	―	149.8, C	―	149.8, C	―	149.3, C	―	148.7, C
9	7.81 (d, 8.5)	115, CH	7.82 (d, 8.0)	115.1, CH	―	125.4, C	―	124.3, C
10	7.46 (t, 8.0)	124.8, CH	7.49 (t, 8.0)	125.2, CH	―	131.4, C	―	131.9, C
11	8.08 (d, 7.5)	127.5, CH	8.10 (d, 8.0)	127.7, CH	8.10 (s)	126.3, CH	8.23 (s)	127.8, CH
12	―	121.3, C	―	121.6, C	―	119.1, C	―	120.8, C
13	―	139.4, C	―	139.2, C	―	137.3, C	―	136, C
14	―	165.6, C	―	165.4, C	―	164.5, C	―	164.5, C
15	4.06 (s)	52.4, CH_3_	4.06 (s)	52.5, CH_3_	4.04 (s)	52.6, CH_3_	―	―
9-Me	―	―	―	―	2.69 (s)	13.7, CH_3_	2.69 (s)	13.6, CH_3_
13-NH	―	―	―	―	11.65 (s, w)	―	10.27 (s)	―

Mycemycins B (**2**) was obtained as a white solid. Its molecular formula was established as C_15_H_10_ClNO_4_ according to the [M + H]^+^ peak at *m*/*z* 304.0378 (calcd for C_15_H_11_ClNO_4_, 304.0377) in the HR-ESI-MS spectrum combined with its ^13^C-NMR data, corresponding to 11 degrees of unsaturation. Analyses of its ^1^H and ^13^C NMR data (Table 1, Figure S3) revealed similar structural features to those of **1**. The most obvious differences between **1** and **2** lay in the positions from C3 to C5. The splitting pattern of H3 changed from “dd” in **1** (coupled with H4 and H5) to “d” in **2** (coupled only with H5). Equally, that of H5 also changed from “dt” in **1** (coupled with H3, H4 and H6) to “dd” in **2** (coupled with H3 and H6). All the changes were due to the lack of a hydrogen atom at the C4 position, which was supported by the DEPT and HSQC correlations (Figure 2 and Figure S3), and by the chemical shift of C4 that changed from δ 119.6 in **1** to δ 124.5 in **2**. The exhibited HMBC signals from H6 (strong signal), H3 (moderate signal) and H5 (weak signal) to the quaternary carbon-C4 further confirmed this deduction (Figure 2 and Figure S3). According to the suggested molecular formula, a Cl- atom should be assigned to C4.

Mycemycins C (**3**) was obtained as a white solid. Its molecular formula was established as C_16_H_11_Cl_2_NO_4_ according to the [M + H]^+^ peak at *m*/*z* 352.0137 (calcd for C_16_H_12_Cl_2_NO_4_, 352.0143) in the HR-ESI-MS spectrum combined with its ^13^C-NMR data, corresponding to 11 degrees of unsaturation. The ^1^H and ^13^C NMR data (Table 1, Figure S4) revealed its similar structural features to those of **2**, especially that the coupling and chemical shifts of H3-H6, C1-C8 and C13-C15 were in good accordance, which were confirmed by the COSY and HMBC correlations (Figure 2, Figure S4). Major differences lay in the positions from C9 to C12. The NMR data of CH (δ_H_ 8.10/δ_C_ 126.3, confirmed by HSQC, Figure S4), which showed accordance with C11 of **1** and **2**, suggested that this carbon was at position 11, too. However, the strong “s” peak of H11 in the ^1^H-NMR spectrum indicated the absence of hydrogen atoms at both C10 and C12 compared with **1** and **2**. According to the HMBC spectrum, the strong correlation signals from 9-Me to C8, C9 and C10 confirmed the connection of C9 and 9-Me; signals from H11 to C12 (weak signal, Figure S4), C13 and C14, and from NH to C12, combined with the chemical shifts of C12 (δ 119.1) and C13 (δ 137.3), confirmed the connections between C11 and C12, C12 and C14, and between NH and C13. The chemical shift of NH (δ 11.65), which revealed the hydrogen bond between 13-NH and 14-carbonyl oxygen atom, also supported the connection between C12 and C14. A further HMBC signal found from H11 to C9, combined with the correlations described above and the chemical shifts from C8 to C13, revealed the overall perspective of the aromatic system. Finally, according to the suggested molecular formula, the second Cl- atom should be assigned to the idle C10 position.

Mycemycins D (**4**) was obtained as a white solid. Its molecular formula was established as C_15_H_11_ClN_2_O_3_ according to the [M + H]^+^ peak at *m*/*z* 303.0538 (calcd for C_15_H_12_ClN_2_O_3_, 303.0536) in the HR-ESI-MS spectrum combined with its ^13^C-NMR data, corresponding to 11 degrees of unsaturation. Its structural fragment from C1 to C7 was identical with that of **1,** well supported by the similarities in their ^1^H and ^13^C NMR data and 2D-NMR correlations (Table 1, Figure 2 and Figure S5). The structural fragment from C8 to C14 was similar to that of **3**, sustained by the NMR data, especially by the similar HMBC correlations from positions 8 to 14, albeit that the chemical shift of H11 in **4** (δ 8.23) was slightly different from that in **1**–**3** (δ 8.08–8.10). Comparisons of the ^1^H and ^13^C NMR spectra between **4** and **1**–**3** revealed the absence of 15-OMe (δ*_H_* 4.04–4.06*/*δ*_C_* 52.4–52.6 in **1**–**3**) in **4** and that, considering the suggested molecular formula, C14 should be connected to an -NH_2_ group.

Mycemycins E (**5**) was obtained as a white solid. Its molecular formula was established as C_15_H_10_Cl_2_N_2_O_3_ according to the [M + H]^+^ peak at *m*/*z* 337.0149 (calcd for C_15_H_11_Cl_2_N_2_O_3_, 337.0146) in the HR-ESI-MS spectrum. This purified compound did not dissolve in any common solvents such as methanol, acetone, acetonitrile, benzene, chloroform, cyclohexane, dichloromethane, dimethylformamide, dimethylsulfoxide, ethyl acetate, formamide, *n*-hexane, pyridine, tetrahydrofuran and toluene, thus, the NMR analysis could not be applied, and no desirable crystals could be obtained for X-ray analysis either. Therefore, its chemical structure (Figure 1) was suggested mainly according to the highly similar MS/MS pattern with that of **3** (Figure S6) and the fact that co-production of **3**–**5** is a biogenetic consideration.

### 2.3. Bioactivities of Compounds **1**–**4**

Mycemycins A–D (**1**–**4**) were evaluated for their antimicrobial activity. These compounds turned out to be inactive against microbes *Staphyloccocus aureus*, *Escherichia coli*, *Bacillus subtilis*, *Mycobacterium gilvum*, *Micrococcus luteus*, *Pseudomonas aeruginosa*, *Fusarium oxysporum* and *Trichoderma viride*. The bioactivity of compounds **1**–**4** were further predicted using Reverse Docking Method [16], and the results indicated potential anti-HIV-1 reverse transcriptase (RT) activity of the compounds. However, because mycemycins had very poor solubility in the HIV-1 RT enzyme reaction buffer and precipitated out before the enzyme was added, their HIV-1 RT inhibitory activity could not be properly measured.

### 2.4. Discussion

Mycemycins are structurally classified as dibenzoxazepinones with two aromatic ring systems. Dibenzoxazepinones have been reported to have various bioactivities and be potential antioxidants [17], calcium channel antagonists [18,19], HIV-1 RT inhibitors [20], *etc*. Most of these compounds are artificially designed and synthesized, with only two natural compounds derived from leaves of herbaceous plant *Carex distachya* [17]. This is the first report of natural dibenzoxazepinones discovered from the microbial kingdom. Interestingly, mycemycins A and B were derived from a terrestrial streptomycete recovered from red soil in China and mycemycins C–E from a marine streptomycete isolated from deep seawater in south Indian Ocean, and these two strains belong to different *Streptomyces* species with a 96.5% 16S rRNA gene sequence similarity. These observations indicate a wide distribution of mycemycins. Moreover, all the marine-derived mycemycins are mono/bis-chlorinated, underpinning that the ocean is a promising source for the discovery of halogenated natural products [21].

GntR-like regulators are often located adjacent to the genes that they control, but there are many exceptions as well [11]. Although the regulatory gene *orf-1741* is flanked by putative secondary metabolic genes, it is impossible in this study to predict the corresponding metabolites that the regulator may involve based on the genome survey sequences. As the halogenase genes (*orf-1802* and *orf-1805*) in FXJ8.012 genome are located relatively far from *orf-1741* (around 50 kb), our results suggest that the GntR encoded by *orf-1741* is likely to be an orphan regulator [11] or an upstream repressor of mycemycin biosynthesis in *S. olivaceus* FXJ8.012. Further efforts need to be made to verify this inference. More experiments are also required to enhance the solubility of mycemycins, especially compound **5**, to better evaluate their bioactivities.

## 3. Experimental Section

### 3.1. General

UV spectra were recorded on a Beckman Coulter DU800 UV/Vis Spectrophotometer, 200 to 400 nm. IR spectra were recorded on a Thermo Nicoiet 8700 spectrometer. HR-ESI-MS spectra were obtained on a Waters Xevo G2 QTOF mass spectrometer with an ACQUITY UPLC BEH C18 column (2.1 mm × 50 mm, 1.7 µm). NMR spectra were measured on a Bruker AV 500 NMR spectrometer. The concentration was performed on an EYELA N-1100S-W rotary evaporator. The analytical and semi-preparative HPLC were both performed on a Shimadzu SPD-M20A HPLC system with a Waters Xbridge ODS column (4.5 × 150 mm, 5 μm, for analysis) and a Sigma-aldrich Ascentis RP-Amide column (4.6 × 150 mm, 5 μm, for semi-preparation). Column chromatography was carried out on silica gel (100–200 mesh) (Qingdao Haiyang Chemical Group Corp., Qingdao, China) and Sephadex LH-20 (Pharmacia).

### 3.2. Strains, Plasmids and Media

Bacterial strains, plasmids and primers used in this study are listed in Tables S1,2 in the supplemental material.

*S.* sp. FXJ1.235 was isolated from an acidic red soil sample collected from Boyang lake, Jiangxi Province, China. The isolation medium was M6 agar and the growth medium was M11 agar [22]. The strain was identified by morphology and 16S rRNA gene sequence analysis using regular procedures [23]. Its 16S rRNA gene sequence (GenBank accession number KJ152035) showed the highest similarity (99%) to *Streptomyces* *galbus* DSM 40089^T^ (GenBank accession number NR_026178). Detailed information of *S. olivaceus* FXJ8.012 (16S rRNA gene sequence HQ622478) was reported previously [9]. *Escherichia coli* Top 10 was used as a host strain for general cloning, and *E. coli* ET12567/pUZ8002 was used for conjugation between *E. coli* and streptomycetes.

GYM agar medium [9] was used for sporulation of strains FXJ1.235 and FXJ8.012Δ*1741*; GYM liquid medium was used for seed culture of strains FXJ1.235 and FXJ8.012Δ*1741*; GYM liquid medium with 1 g/L NaCl was used as the fermentation medium for FXJ1.235; modified R2 medium [15] was used as the fermentation medium for FXJ8.012Δ*1741*. LB and PDA agars were used for antibacterial and antifungal tests, respectively, as previously described [9]. *E. coli* strains carrying plasmids for nucleic acid manipulation were cultured in liquid or agar LB medium, added with ampicillin (100 μg/mL), nalidixic acid (25 μg/mL), apramycin (50 μg/mL), chloramphenicol (25 μg/mL) or kanamycin (100 μg/mL) as appropriate.

### 3.3. Nucleic Acid Manipulations and Mutant Construction

The DNA sequence of *orf-1741* has been deposited in the GenBank database with an accession number KT357292.

Nucleic acid manipulations for *E. coli* and streptomycetes were performed according to standard approaches [24,25]. Inactivation of *orf-1741* was performed by gene replacement via double-crossover homologous recombination. The kanamycin resistance gene *neo* was obtained by amplification from a recombinant plasmid pUC119::*neo* with primer pairs *neo*-*EcoRI*-Forward and *neo*-*EcoRI*-Reverse (Table S2) [26]. Upstream and downstream regions of approximately 2 kb flanking *orf-1741* were amplified by PCR with primer pairs *1741*-L-Forward/*1741*-L-Reverse and *1741*-R-Forward/*1741*-R-Reverse (Table S2). These PCR products were digested with *Hin*dIII/*Eco*RI or *Xba*I/*Eco*RI, then separated by agarose gel electrophoresis and purified from the gel. The resulting fragments were ligated with the *Eco*RI-digested *neo* gene and *Xba*I*-* and *Hin*dIII-digested plasmid pKC1139 to generate pKC1139::*1741*::*neo*, which was subsequently introduced into *S. olivaceus* FXJ8.012 by conjugation via ET12567/pUZ8002. Spores of exconjugants were harvested and spread on MS agar [25] containing kanamycin. After incubation at 38 °C for a week, apramycin-sensitive and kanamycin-resistant colonies were identified, and further verified as *orf-1741* disruption mutants (Δ*1741*) by PCR with primer pairs Δ*1741*-Foward and Δ*1741*-Reverse (Figure S7) and sequencing of the resulting amplimers.

### 3.4. Fermentation, Extraction and Isolation

#### 3.4.1. *S.* sp. FXJ1.235

After growth on GYM agar in petri dishes at 28 °C for 7 days, a suitable amount of spores of *S.* sp. FXJ1.235 were transferred into a 500-mL shake flask containing 100 mL of GYM liquid medium. The resulting culture was incubated on a rotary shaker at 170–180 rpm and 28 °C for 5 days to afford a seed culture, which was used to inoculate 100 shake flasks (500 mL) each containing 100 mL of GYM liquid medium supplemented with NaCl. The inoculated flasks were incubated under similar conditions for 7 days.

After fermentation, the culture broth (10 L) was centrifuged at 5000 rpm for 15 min to separate mycelia and supernatant. The resulting mycelium cake was extracted three times with an equal volume of ethanol for 8 h. The ethanol extracts were combined and concentrated *in vacuo*, and then subjected to column chromatography on silica gel (100–200 mesh) using acetone as the elution solvent to obtain a mixture of crude products (1.1–1.2 g). The mixture was passed repeatedly through a Sephadex LH-20 column eluting with MeOH/CHCl_3_ (4:1) to remove fatty acids, nucleosides and other impurities. Final purification was carried out by RP-HPLC using a Sigma-aldrich Ascentis RP-Amide column eluting with acetonitrile/H_2_O (7:3) to yield **1** (7.1 mg) and **2** (5.8 mg).

#### 3.4.2. *S. olivaceus* FXJ8.012

The inoculation and fermentation process for FXJ8.012Δ*1741* was almost the same as that for FXJ1.235. Differences lay in the large scale fermentation where 200 shake flasks (500 mL) each containing 100 mL of modified R2 medium [15] were used for FXJ8.012Δ*1741*.

The separation and purification procedures for the metabolites of FXJ8.012Δ*1741* were almost identical with those for **1** and **2**. About 5.1 g crude products were obtained after silica gel column chromatography; and in the final RP-HPLC procedure, the mixture of compounds was eluted with acetonitrile/H_2_O (76:24) to yield **3** (1.3 mg), **4** (0.8 mg) and **5** (about 5–6 mg).

Mycemycin A (**1**): white solid; UV (MeOH) λ_max_ 244, 262, 272(sh), 292(sh), 305, 332, 345(sh) nm; IR (KBr) ν_max_ 2954, 2917, 1716, 1634, 1599, 1549, 1488, 1426, 1305, 1259, 1244, 1192, 1160, 1140, 1061, 1013, 939, 876, 787, 753, 709, 537, 493, 480 cm^−1^; ^1^H- and ^13^C-NMR data, Table 1; HR-ESI-MS m/z 270.0776 [M + H]^+^ (calcd for C_15_H_12_NO_4_, 270.0766).

Mycemycin B (**2**): white solid; UV (MeOH) λ_max_ 244, 265, 275(sh), 295, 307, 340, 351(sh) nm; IR (KBr) ν_max_ 2954, 2921, 1716, 1631, 1584, 1541, 1479, 1425, 1281, 1242, 1194, 1134, 1060, 1022, 946, 879, 822, 788, 759, 724, 547, 487 cm^−1^; ^1^H- and ^13^C-NMR data, Table 1; HR-ESI-MS m/z 304.0378 [M + H]^+^ (calcd for C_15_H_11_ClNO_4_, 304.0377).

Mycemycin C (**3**): white solid; UV (MeOH) λ_max_ 251, 259(sh), 269(sh), 279(sh), 295(sh), 307(sh), 333, 345(sh) nm; ^1^H- and ^13^C-NMR data, Table 1; HR-ESI-MS m/z 352.0137 [M + H]^+^ (calcd for C_16_H_12_Cl_2_NO_4_, 352.0143).

Mycemycin D (**4**): white solid; UV (MeOH) λ_max_ 244(sh), 250, 260(sh), 273(sh), 285(sh), 297(sh), 312, 344, 356(sh) nm; ^1^H- and ^13^C-NMR data, Table 1; HR-ESI-MS m/z 303.0538 [M + H]^+^ (calcd for C_15_H_12_ClN_2_O_3_, 303.0536).

Mycemycin E (**5**): white solid; UV (MeOH) λ_max_ 244(sh), 251, 260(sh), 270(sh), 283(sh), 300(sh), 311, 340, 352(sh) nm; HR-ESI-MS m/z 337.0148 [M + H]^+^ (calcd for C_15_H_11_Cl_2_N_2_O_3_, 337.0146).

### 3.5. Bioactivity Assay

Primary antimicrobial assays were conducted using the agar-diffusion method. 50 μg of compound were transferred to a sterile paper disc of 5 mm diameter, which was put onto the surface of LB or PDA agar containing pathogenic bacteria or fungi, followed by incubation at 37 °C overnight for bacteria or 25 °C for 1–2 days for fungi. Bioactivity was determined by observing the inhibition zones.

The assay of HIV-1 RT inhibitory activity was carried out in triplicate using the Reverse Transcriptase Assay, colorimetric kit (Roche 11468120910), following the standard procedure provided by the manufacture. The initial concentration for compounds **1**–**4** was set to 1000 μg/mL, and that for the positive control EFV to 1000 ng/mL.

## 4. Conclusions

In this study, we applied genetic manipulation to a marine-derived streptomycete, leading to the discovery of three new halogenated compounds, mycemycins C–E (**3**–**5)**, which were structurally similar to another two new compounds, mycemycins A and B (**1** and **2)** isolated from a soil-derived streptomycete. These new compounds were elucidated as dibenzoxazepinone derivatives, and are the first dibenzoxazepinones discovered from microbial source. These findings suggest that marine-derived actinomycetes have great potential to produce novel natural products, notably halogenated ones, as their terrestrial counterparts, and new approaches such as manipulation of regulators are effective at activating the biosynthesis of these cryptic marine compounds.

## Supplementary Files

Supplementary File 1
