# Supplementary material for: Mycemycins A–E, New Dibenzoxazepinones Isolated from Two Different Streptomycetes
Source: Mar Drugs. 2015 Sep 30;13(10):6247–58. doi: 10.3390/md13106247 (PMC4626687; doi:10.3390/md13106247)
Supplement: Supplementary File 1 [file marinedrugs-13-06247-s001.docx]

**Supplementary Information**

| **Figure S1** | HPLC and UHPLC-MS analyses of the fermentation  extracts of WT and Δ*1741* mutant. | S3 |
| --- | --- | --- |
| **Figure S2** | Spectral data for compound **1**. | S4–S8 |
| **(A)** | HR-ESI-MS spectrum of **1**. | S4 |
| **(B)** | UV spectrum of **1**. | S4 |
| **(C)** | IR spectrum of **1**. | S5 |
| **(D)** | ^1^H-NMR spectrum (500 MHz, *CDCl_3_*) of **1**. | S5 |
| **(E)** | ^13^C-NMR spectrum (125 MHz, *CDCl_3_*) of **1**. | S6 |
| **(F)** | DEPT 135 spectrum (125 MHz, *CDCl_3_*) of **1**. | S6 |
| **(G)** | ^1^H–^1^H-COSY spectrum (500 × 500 MHz, *CDCl_3_*) of **1**. | S7 |
| **(H)** | ^1^H–^13^C-HSQC spectrum (500 × 125 MHz, *CDCl_3_*) of **1**. | S7 |
| **(I)** | ^1^H–^13^C-HMBC spectrum (500 × 125 MHz, *CDCl_3_*) of **1**. | S8 |
| **Figure S3** | Spectral data for compound **2**. | S8–S12 |
| **(A)** | HR-ESI-MS spectrum of **2**. | S8 |
| **(B)** | UV spectrum of **2**. | S9 |
| **(C)** | IR spectrum of **2**. | S9 |
| **(D)** | ^1^H-NMR spectrum (500 MHz, *CDCl_3_*) of **2**. | S10 |
| **(E)** | ^13^C-NMR spectrum (125 MHz, *CDCl_3_*) of **2**. | S10 |
| **(F)** | DEPT 135 spectrum (125 MHz, *CDCl_3_*) of **2**. | S11 |
| **(G)** | ^1^H–^1^H-COSY spectrum (500 × 500 MHz, *CDCl_3_*) of **2**. | S11 |
| **(H)** | ^1^H–^13^C-HSQC spectrum (500 × 125 MHz, *CDCl_3_*) of **2**. | S12 |
| **(I)** | ^1^H–^13^C-HMBC spectrum (500 × 125 MHz, *CDCl_3_*) of **2**. | S12 |
| **Figure S4** | Spectral data for compound **3** | S13–S17 |
| **(A)** | HR-ESI-MS spectrum of **3**. | S13 |
| **(B)** | UV spectrum of **3**. | S13 |
| **(C)** | ^1^H-NMR spectrum (500 MHz, *CDCl_3_*) of **3**. | S14 |
| **(D)** | ^13^C-NMR spectrum (125 MHz, *CDCl_3_*) of **3**. | S14 |
| **(E)** | DEPT 135 spectrum (125 MHz, *CDCl_3_*) of **3**. | S15 |
| **(F)** | ^1^H–^1^H-COSY spectrum (500 × 500 MHz, *CDCl_3_*) of **3**. | S15 |
| **(G)** | ^1^H–^13^C-HSQC spectrum (500 × 125 MHz, *CDCl_3_*) of **3**. | S16 |
| **(H)** | ^1^H–^13^C-HMBC spectrum (500 × 125 MHz, *CDCl_3_*) of **3**. | S17 |
| **Figure S5** | Spectral data for compound **4**. | S18–S21 |
| **(A)** | HR-ESI-MS spectrum of **4**. | S18 |
| **(B)** | UV spectrum of **4**. | S18 |
| **(C)** | ^1^H-NMR spectrum (500 MHz, *CDCl_3_*) of **4**. | S18 |
| **(D)** | ^13^C-NMR spectrum (125 MHz, *CDCl_3_*) of **4**. | S19 |
| **(E)** | DEPT 135 spectrum (125 MHz, *CDCl_3_*) of **4**. | S19 |
| **(F)** | ^1^H–^1^H-COSY spectrum (500 × 500 MHz, *CDCl_3_*) of **4**. | S20 |
| **(G)** | ^1^H–^13^C-HSQC spectrum (500 × 125 MHz, *CDCl_3_*) of **4**. | S20 |
| **(H)** | ^1^H–^13^C-HMBC spectrum (500 × 125 MHz, *CDCl_3_*) of **4**. | S21 |
| **Figure S6** | Spectral data for compound **5** | S22–S23 |
| **(A)** | HR-ESI-MS spectrum of **5**. | S22 |
| **(B)** | UV spectrum of **5** (collected with HPLC DAD  detector at MeOH:H_2_O = 4:1). | S22 |
| **(C)** | HR-ESI-MS/MS comparison of **3** and **5**. | S23 |
| **Figure S7** | PCR analysis of Δ*1741* double-crossover mutant. | S23 |
| **Table S1** | Strains and plasmids used in this study. | S24 |
| **Table S2** | Primers used in this study (restriction sites used are underlined; protective nucleotides are in italics). | S24 |
| **References** |  | S25 |


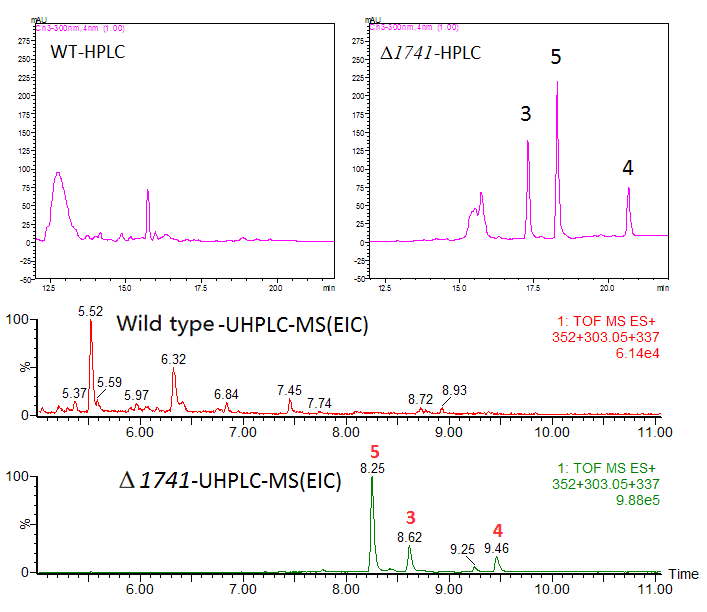


**Figure S1.** HPLC and UHPLC-MS analyses of the fermentation extracts of WT and Δ*1741* mutant. For HPLC analyses, the Waters Xbridge ODS column was used and the
column was eluted as follows: 0.01 min − 80% A + 20% B, 15.00 min − 0% A + 100% B, 20.00 min − 0% A + 100% B, 20.01 min − 80% A + 20% B, 25.01 min − 80% A + 20% B, where A was water and B was methanol. For UHPLC-MS analyses, the ACQUITY UPLC BEH C18 column was used and the column was eluted as follows: 0.00 min − 95% A + 5% B, 10.00 min − 0% A + 100% B, 12.00 min − 0% A + 100% B, 12.01 min − 95% A + 5% B, 14.00 min − 95% A + 5% B, where A was water (0.1% formic acid) and B was acetonitrile.


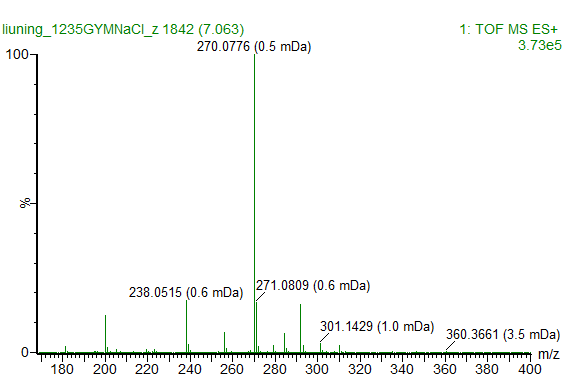


(**A**)

(**B**)

**Figure S2.** *Cont.*


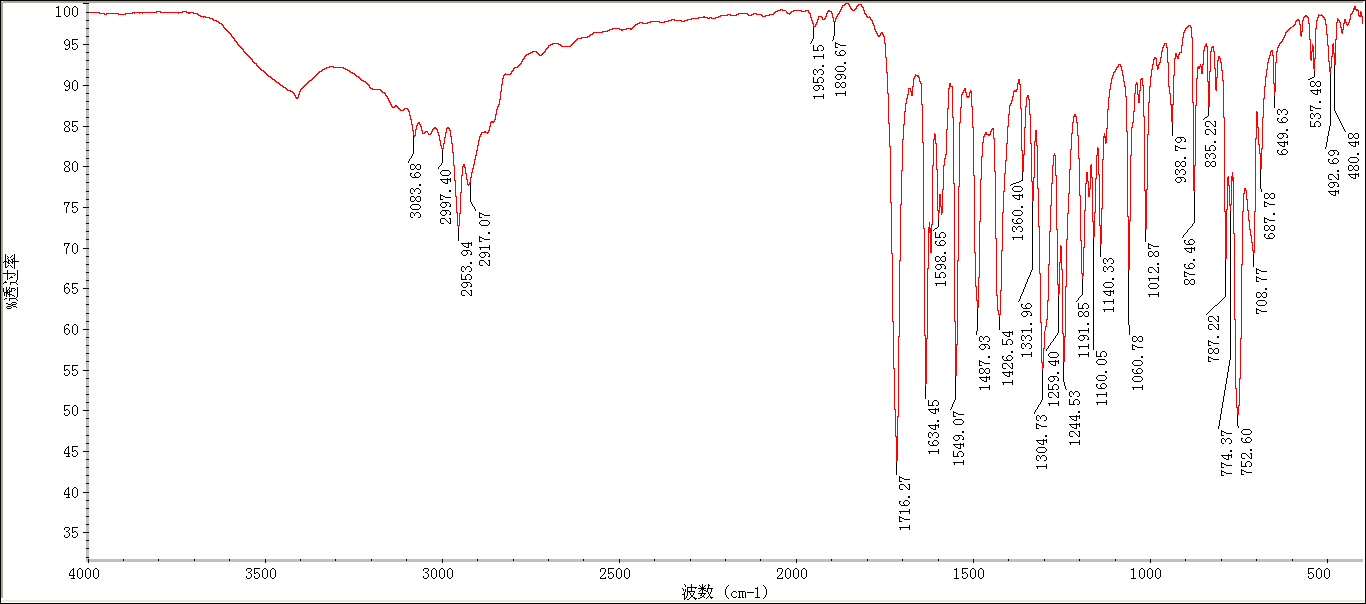


(**C**)


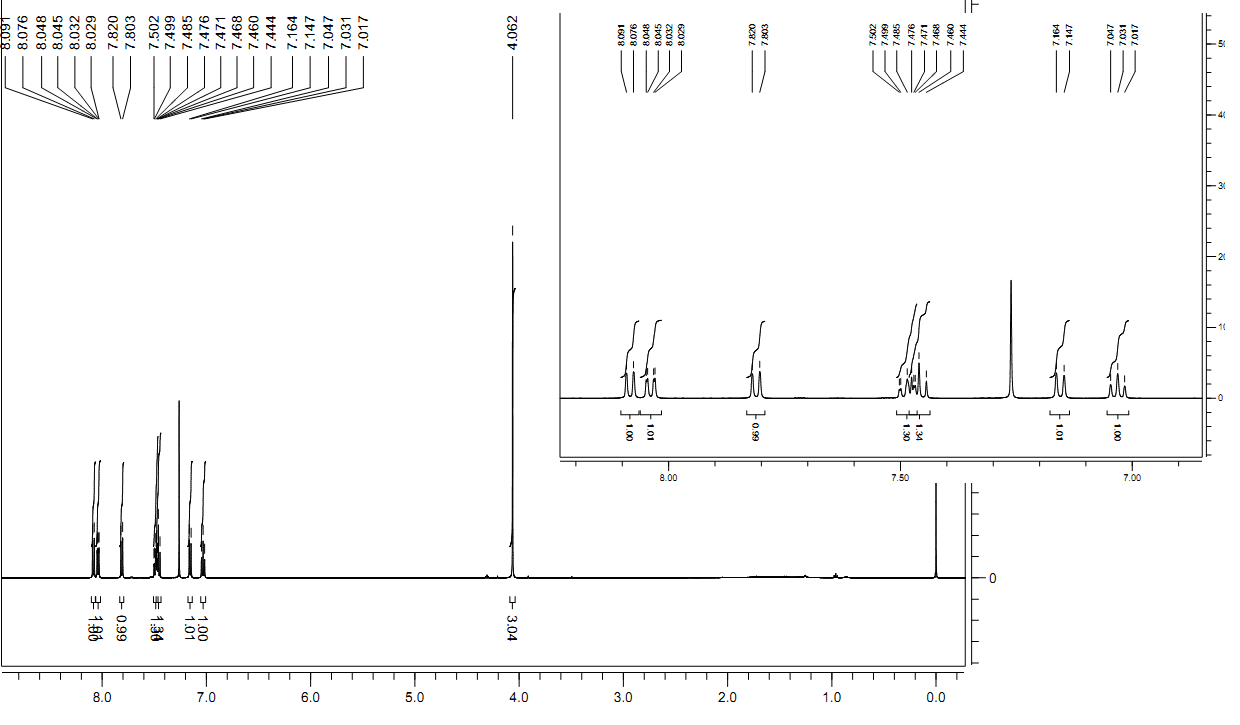


(**D**)

**Figure S2.** *Cont.*


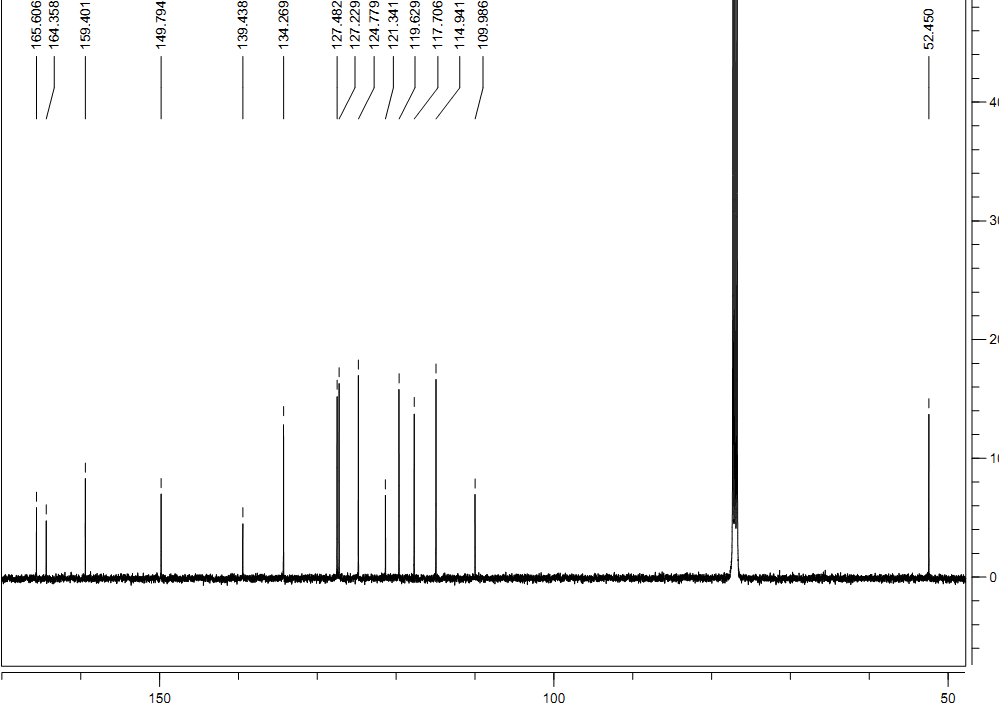


(**E**)


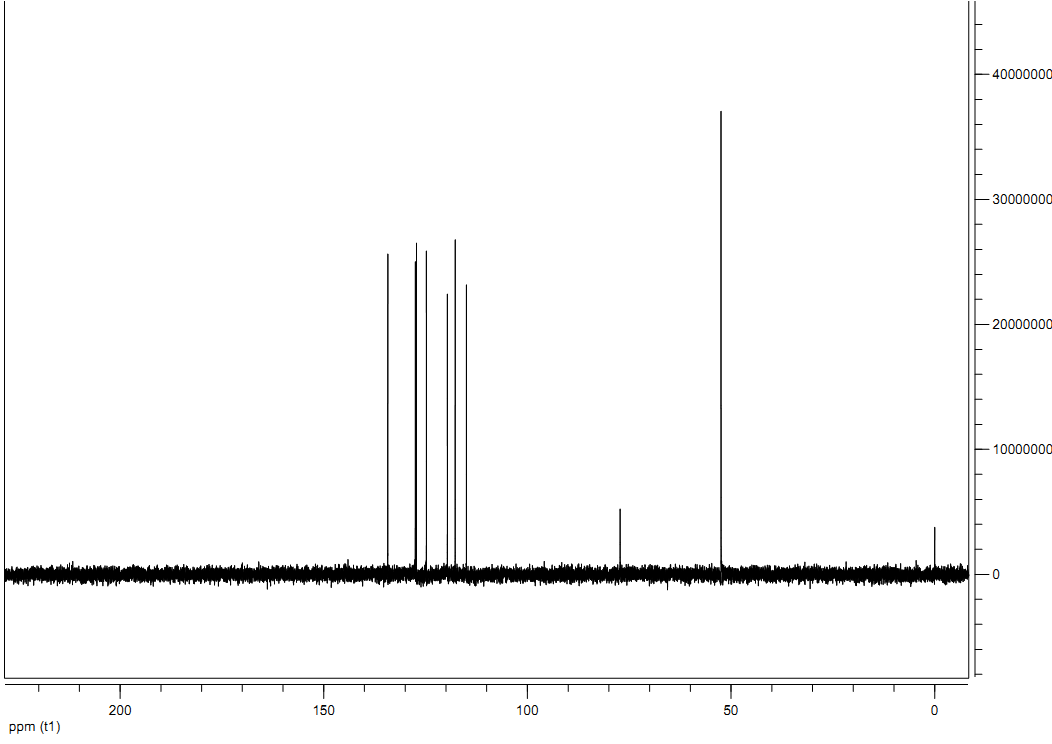


(**F**)

**Figure S2.** *Cont.*


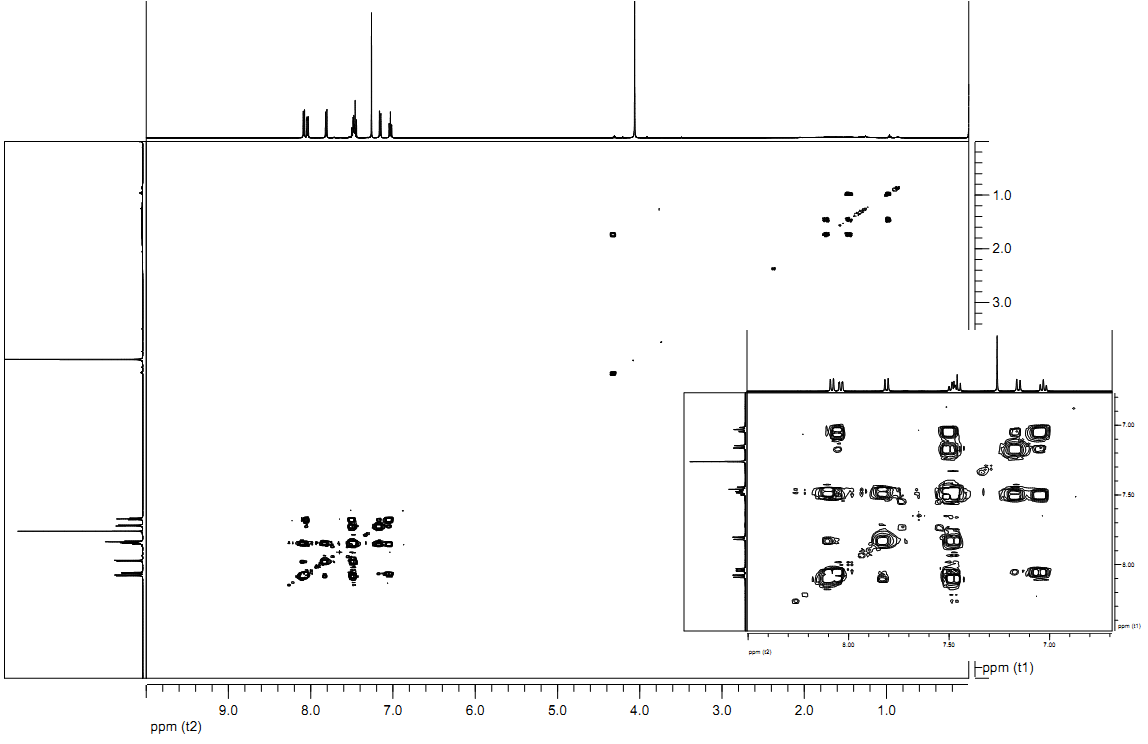


(**G**)


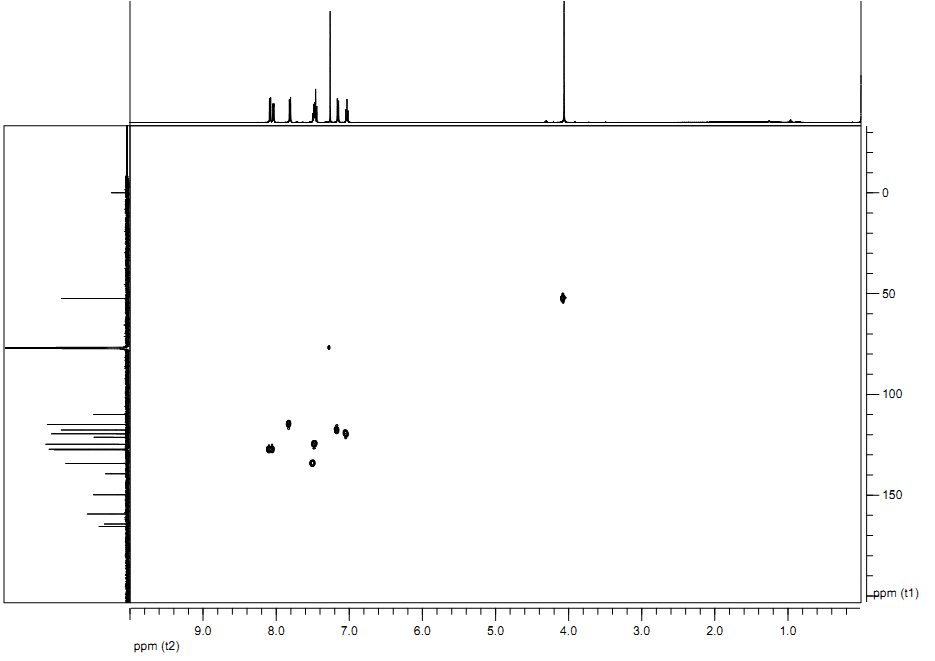


(**H**)

**Figure S2.** *Cont.*


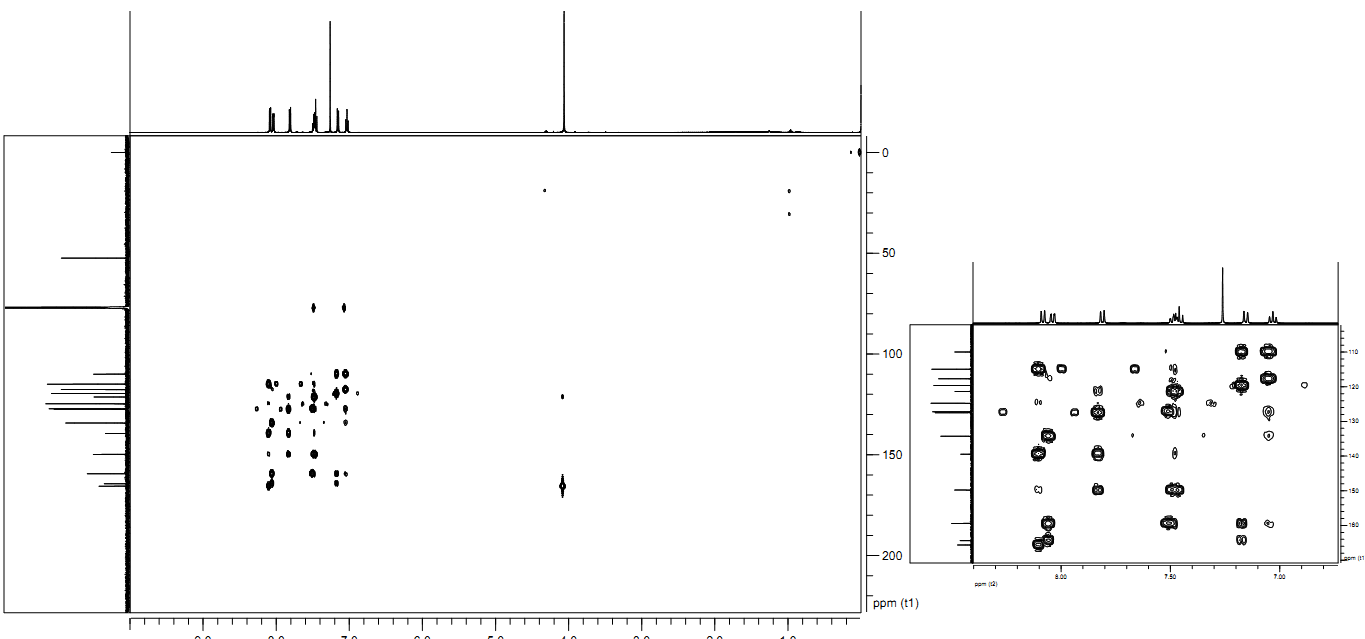


(**I**)

**Figure S2.** (**A**) HR-ESI-MS spectrum of **1**; (**B**) UV spectrum of **1**; (**C**) IR spectrum of **1**;
(**D**) ^1^H-NMR spectrum (500 MHz, *CDCl_3_*) of **1**; (**E**) ^13^C-NMR spectrum (125 MHz, *CDCl_3_*) of **1**; (**F**) DEPT 135 spectrum (125 MHz, *CDCl_3_*) of **1**; (**G**) ^1^H–^1^H-COSY spectrum (500 × 500 MHz, *CDCl_3_*) of **1**; (**H**) ^1^H–^13^C-HSQC spectrum (500 × 125 MHz, *CDCl_3_*) of **1**;
(**I**) ^1^H–^13^C-HMBC spectrum (500 × 125 MHz, *CDCl_3_*) of **1**.


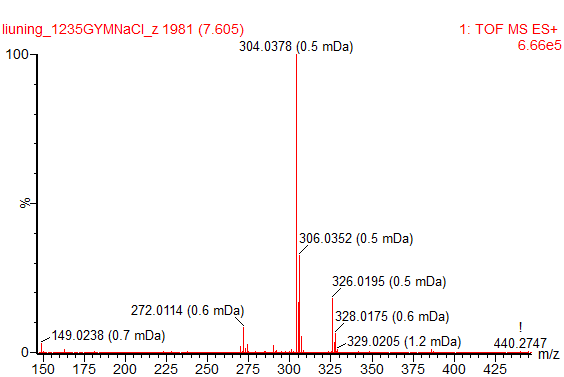


(**A**)

**Figure S3.** *Cont.*

(**B**)


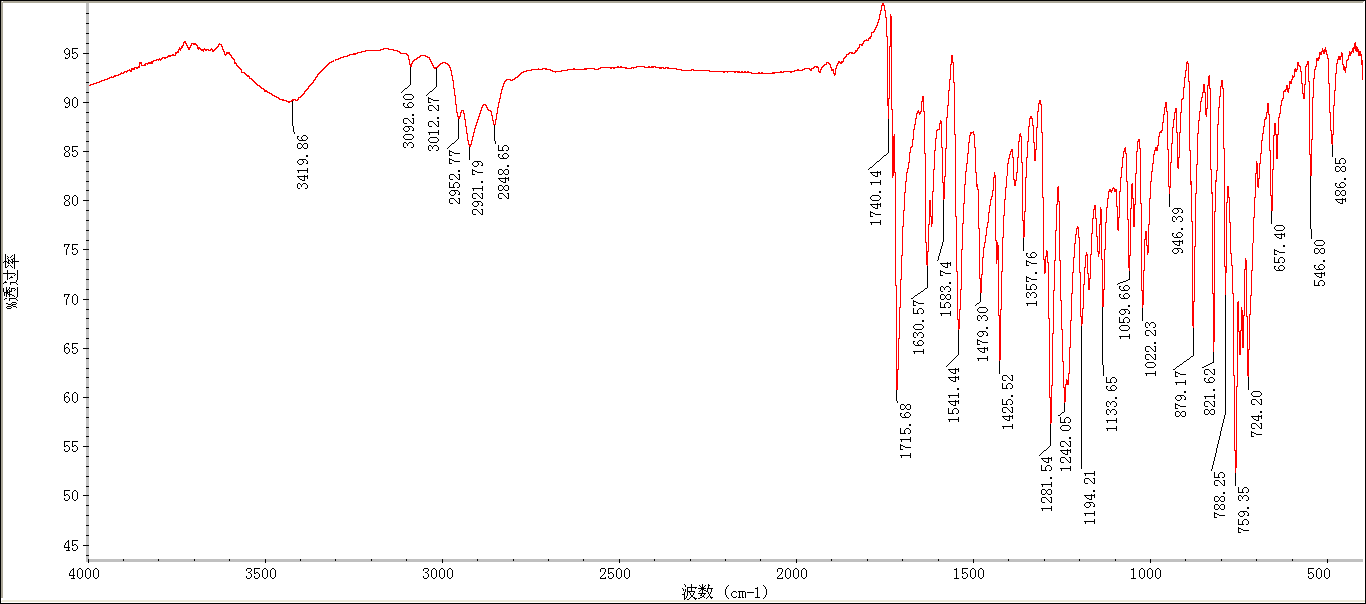


(**C**)

**Figure S3.** *Cont.*


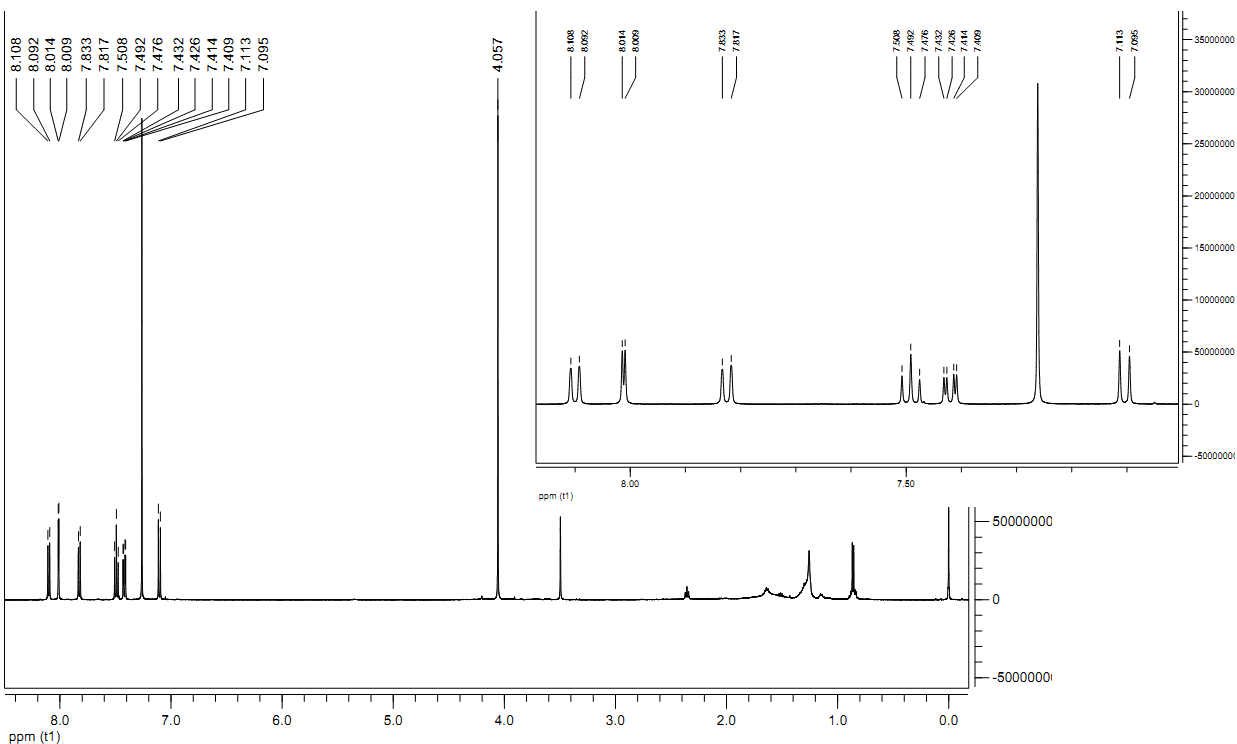


(**D**)


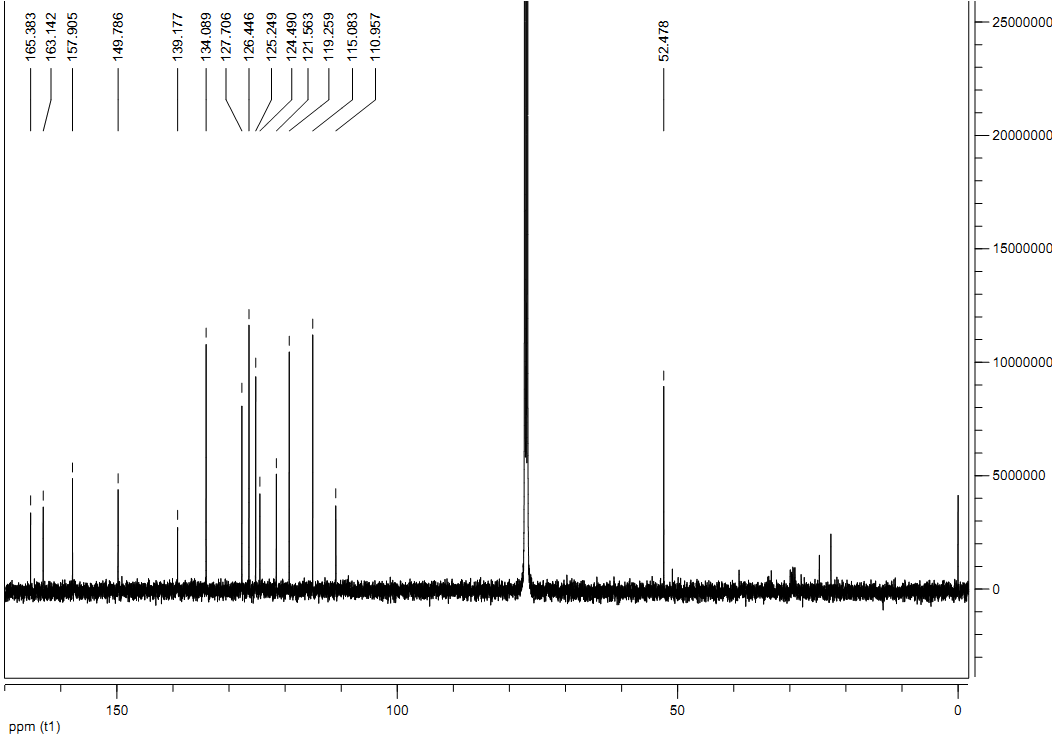


(**E**)

**Figure S3.** *Cont.*


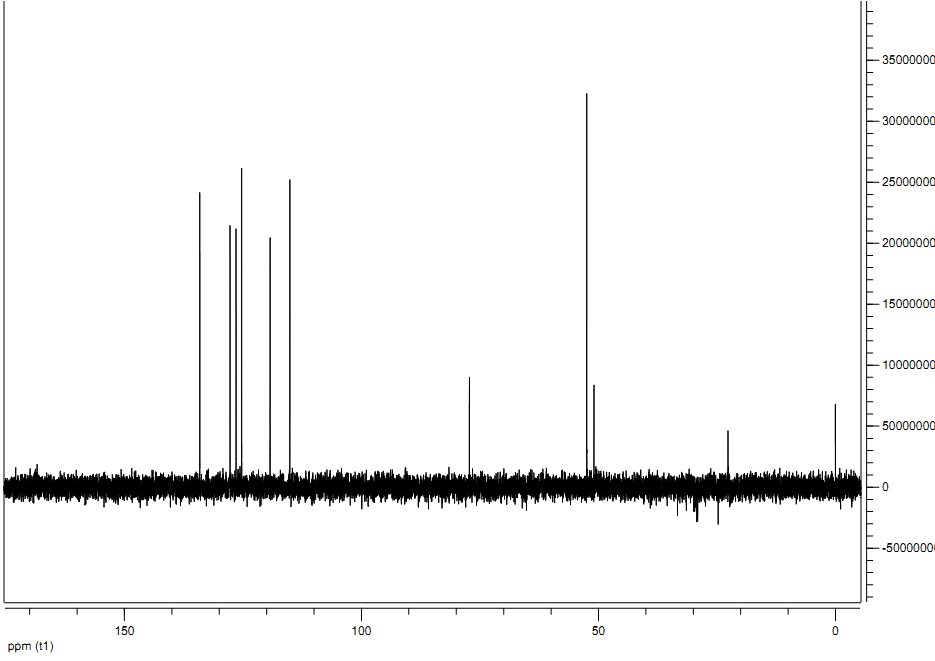


(**F**)


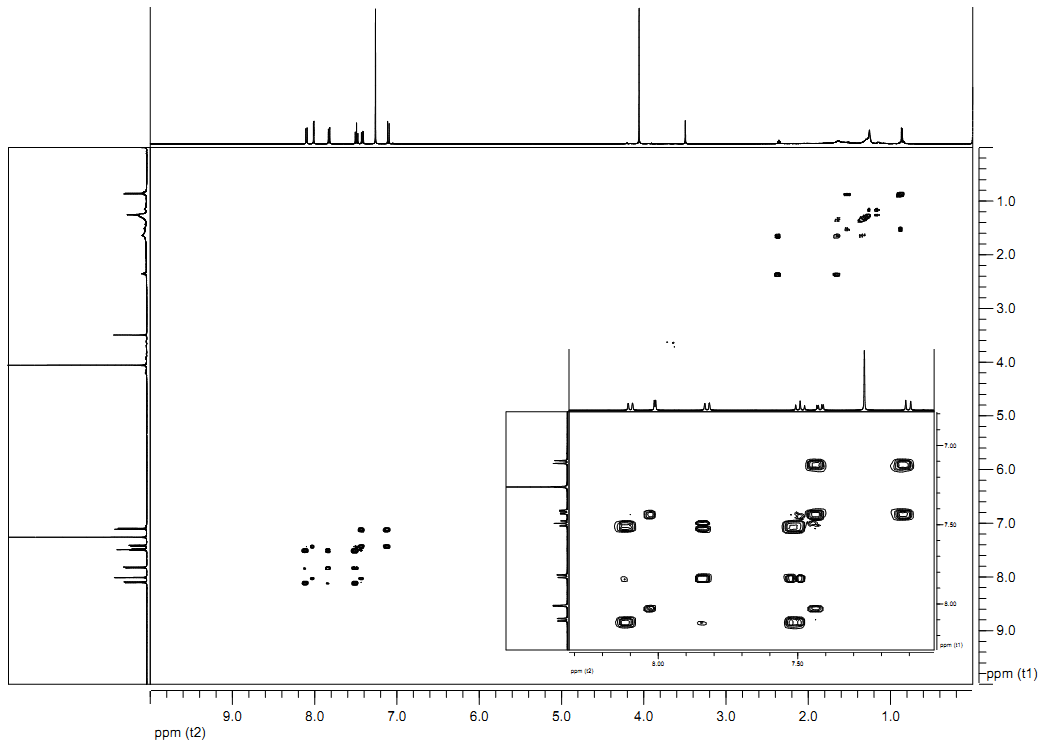


**(G)**

**Figure S3.** *Cont.*


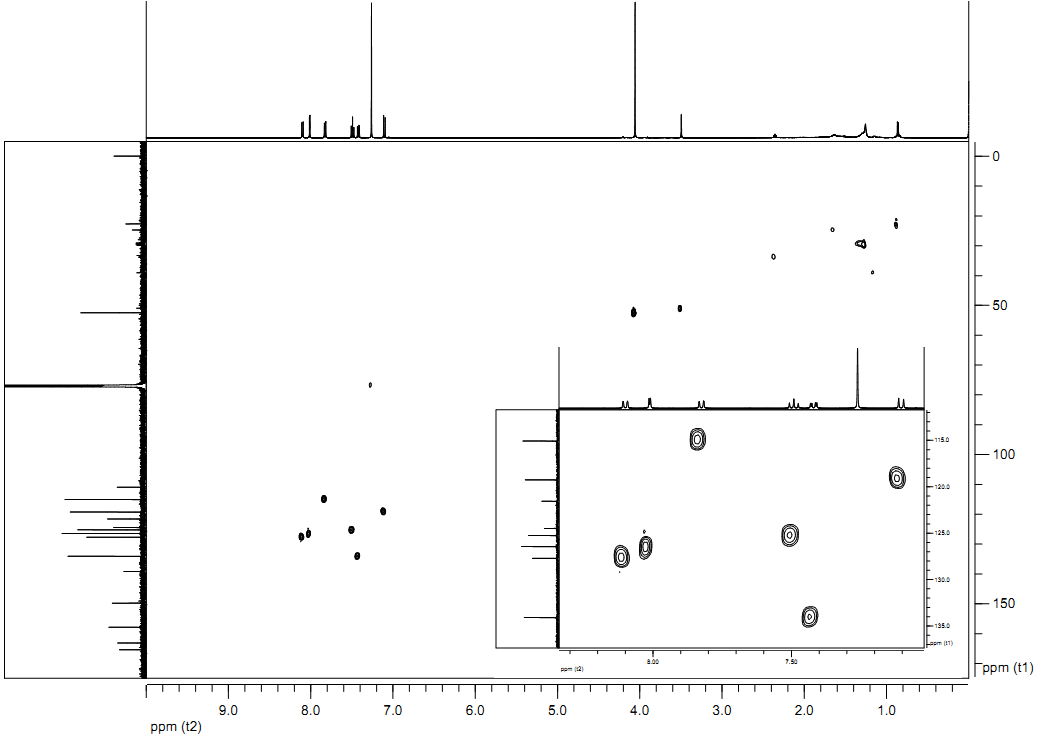


(**H**)


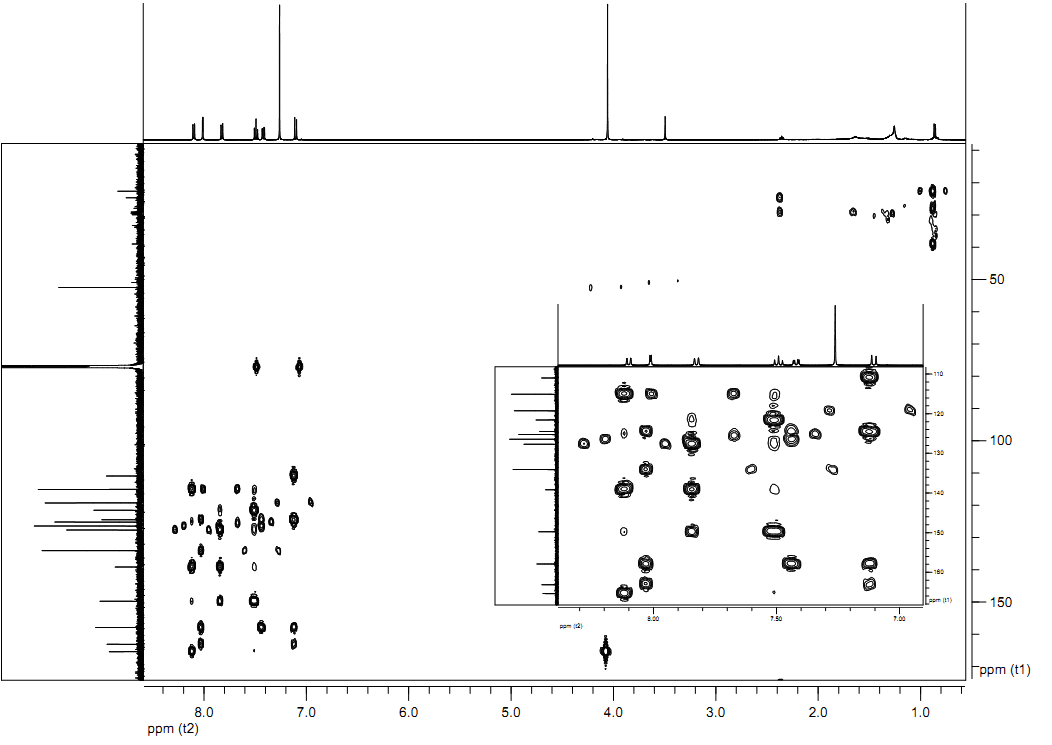


(**I**)

**Figure S3.** (**A)** HR-ESI-MS spectrum of **2**; (**B**) UV spectrum of **2**; (**C**) IR spectrum of **2**;
(**D**) ^1^H-NMR spectrum (500 MHz, *CDCl_3_*) of **2**; (**E**) ^13^C-NMR spectrum (125 MHz, *CDCl_3_*) of **2**; (**F**) DEPT 135 spectrum (125 MHz, *CDCl_3_*) of **2**; (**G**) ^1^H–^1^H-COSY spectrum (500 × 500 MHz, *CDCl_3_*) of **2**; (**H**) ^1^H–^13^C-HSQC spectrum (500 × 125 MHz, *CDCl_3_*) of **2**;
(**I**) ^1^H–^13^C-HMBC spectrum (500 × 125 MHz, *CDCl_3_*) of **2**.

(**A**)

(**B**)

**Figure S4.** *Cont.*


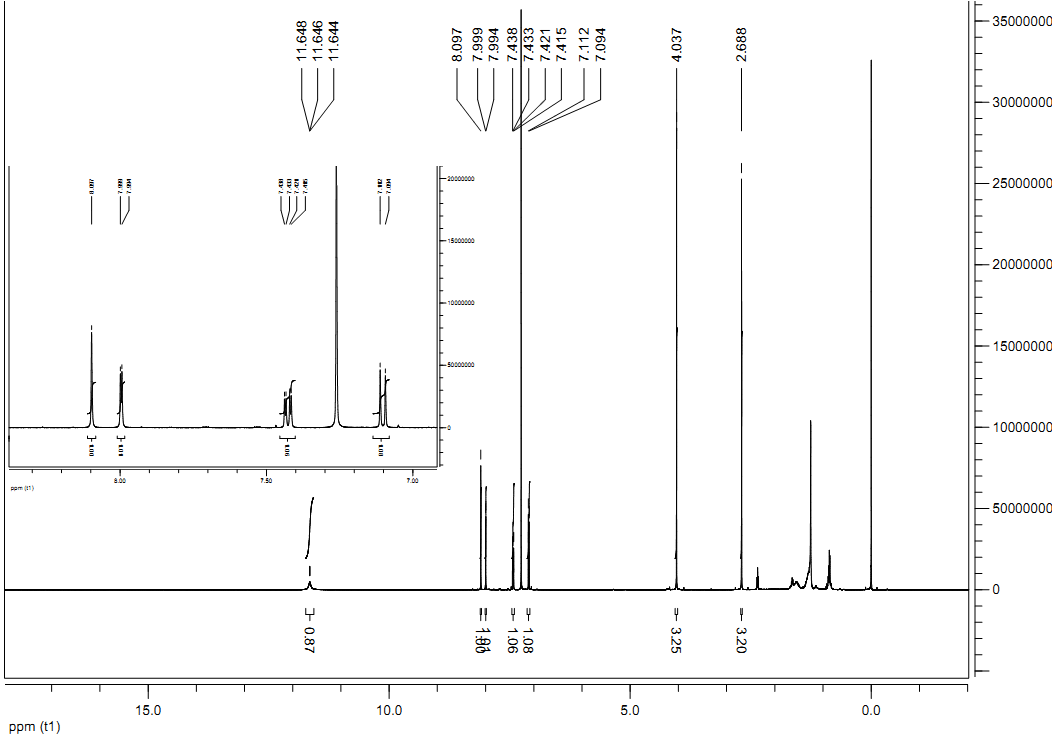


(**C**)


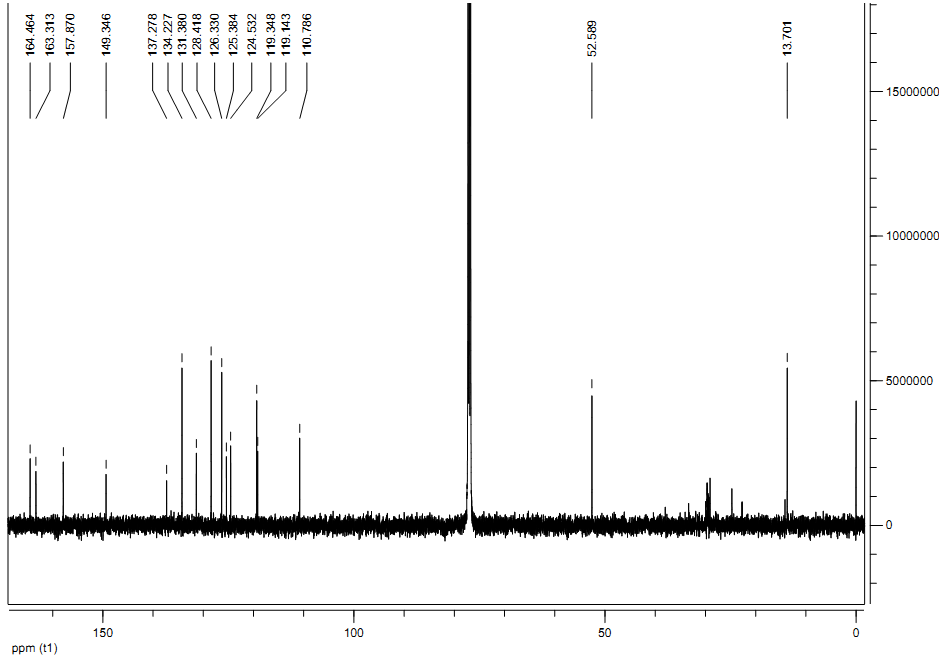


(**D**)

**Figure S4.** *Cont.*


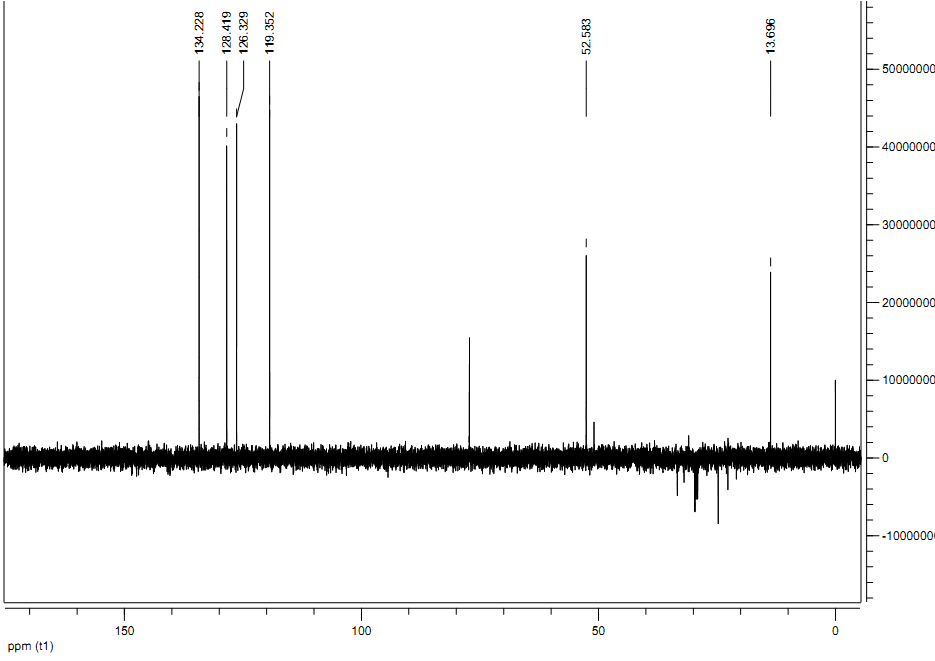


(**E**)


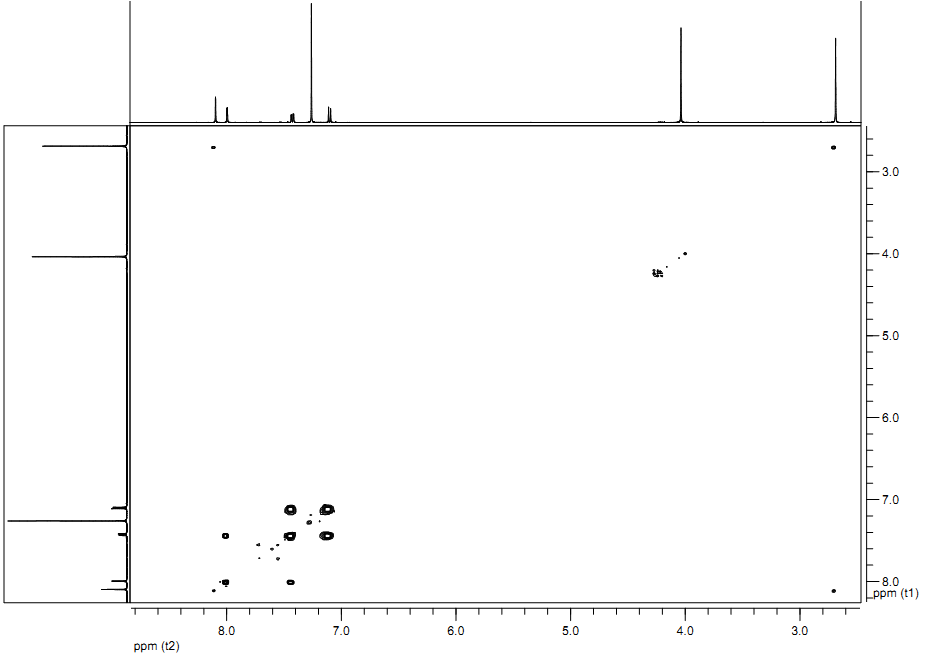


(**F**)

**Figure S4.** *Cont.*


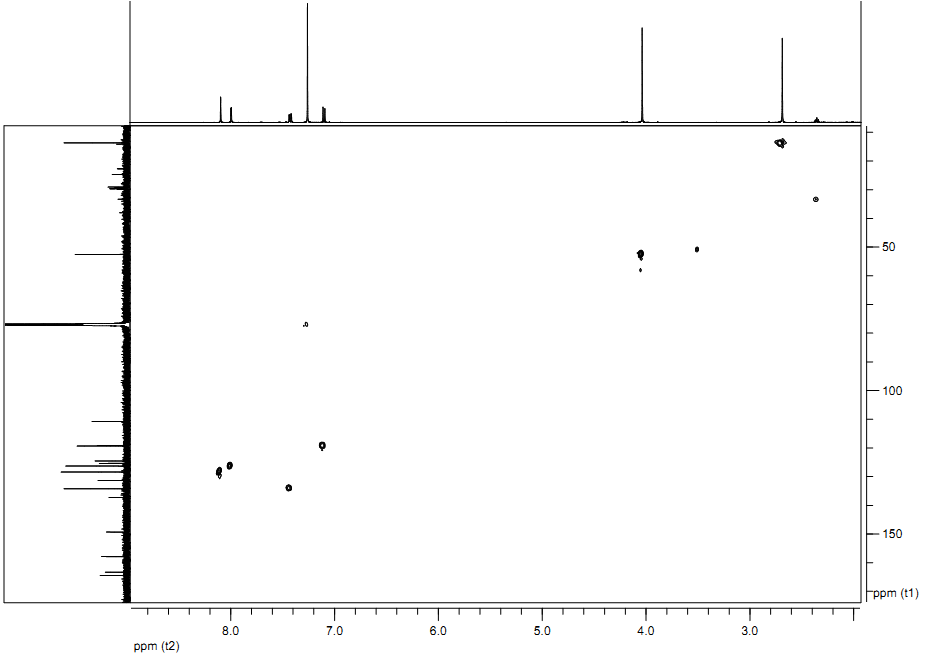


(**G**)

**Figure S4.** *Cont.*


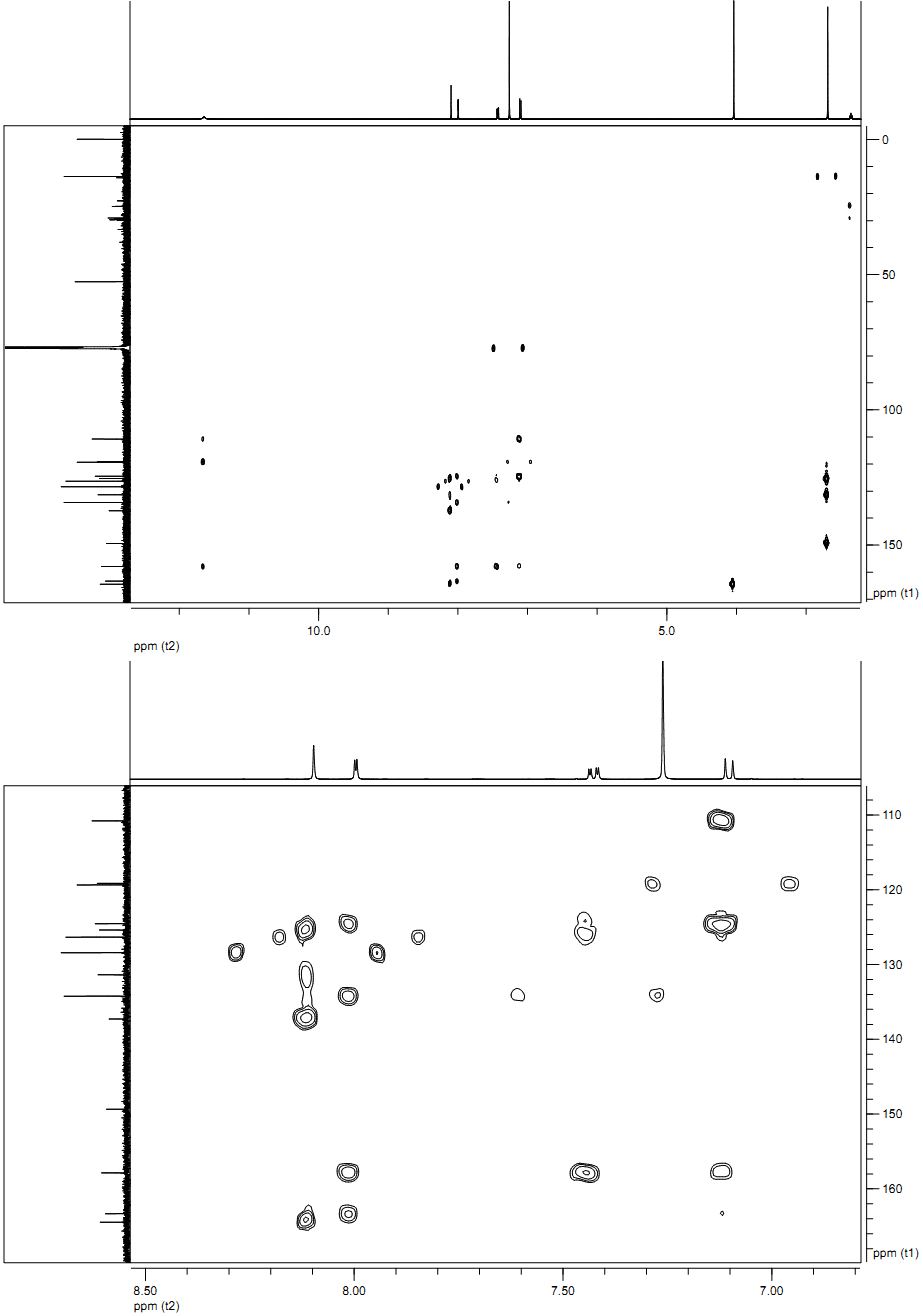


(**H**)

**Figure S4.** (**A**) HR-ESI-MS spectrum of **3**; (**B)** UV spectrum of **3**; (**C**) ^1^H-NMR spectrum (500 MHz, *CDCl_3_*) of **3**; (**D**) ^13^C-NMR spectrum (125 MHz, *CDCl_3_*) of **3**; (**E**) DEPT 135 spectrum (125 MHz, *CDCl_3_*) of **3**; (**F**) ^1^H–^1^H-COSY spectrum (500 × 500 MHz, *CDCl_3_*) of **3**; (**G**) ^1^H–^13^C-HSQC spectrum (500 × 125 MHz, *CDCl_3_*) of **3**; (**H**) ^1^H–^13^C-HMBC spectrum (500 × 125 MHz, *CDCl_3_*) of **3**.

(**A**)

(**B**)


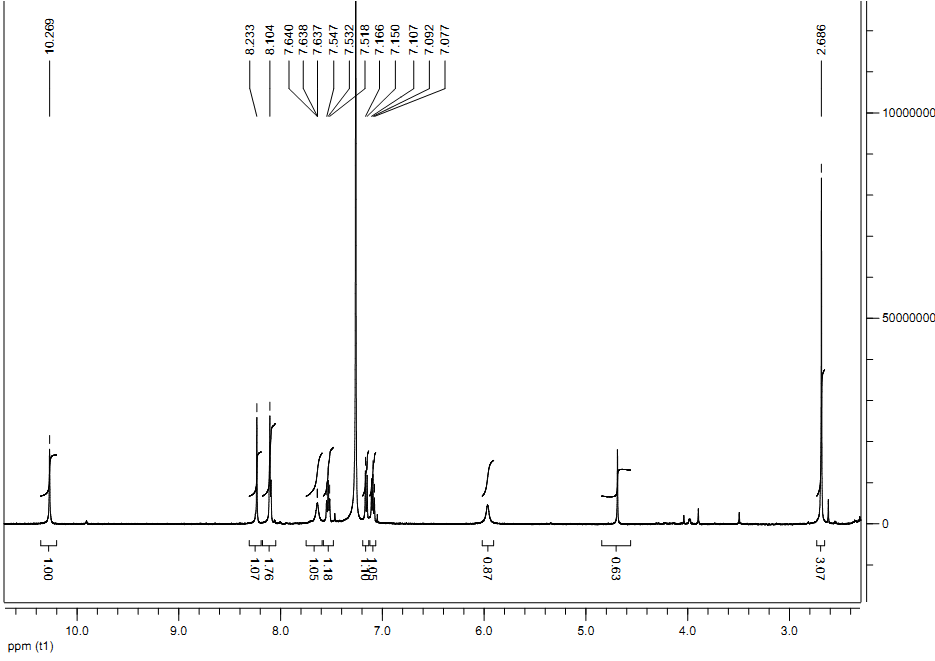


(**C**)

**Figure S5.** *Cont.*


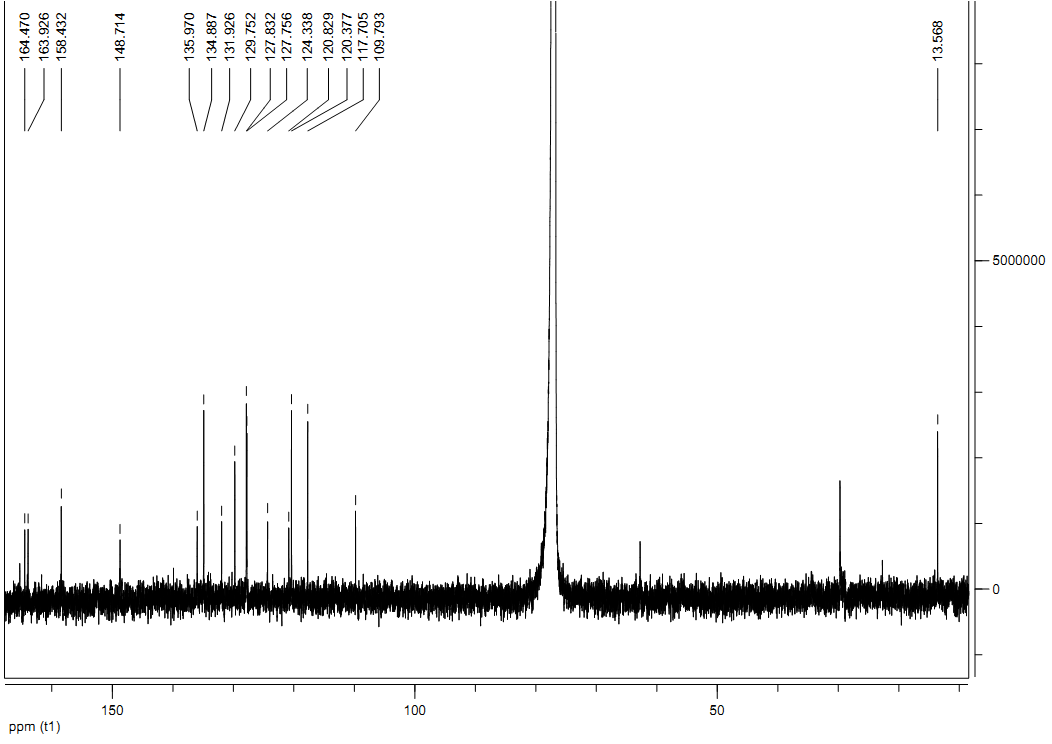


(**D**)


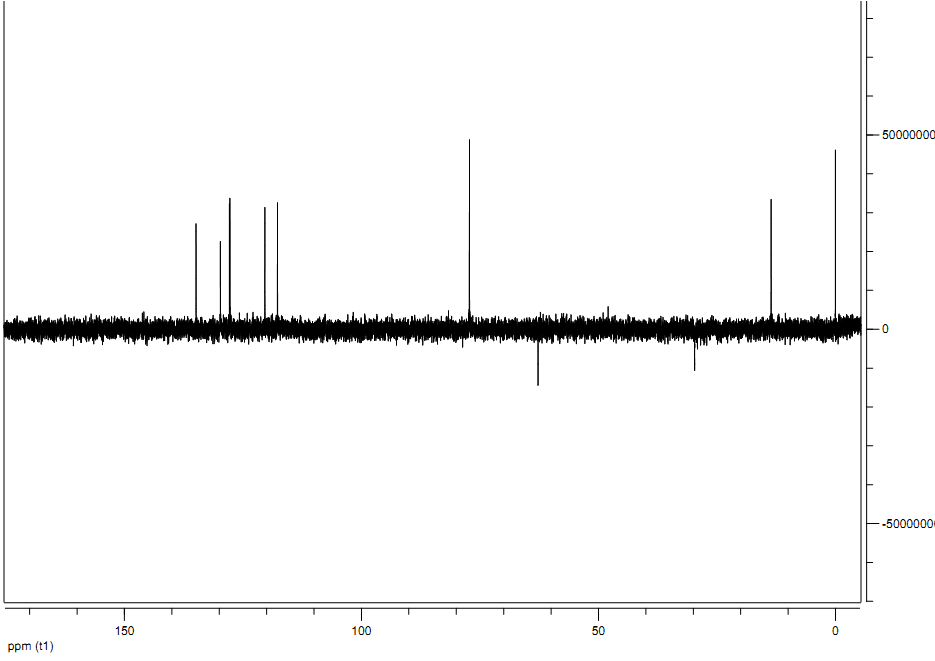


(**E**)

**Figure S5.** *Cont.*


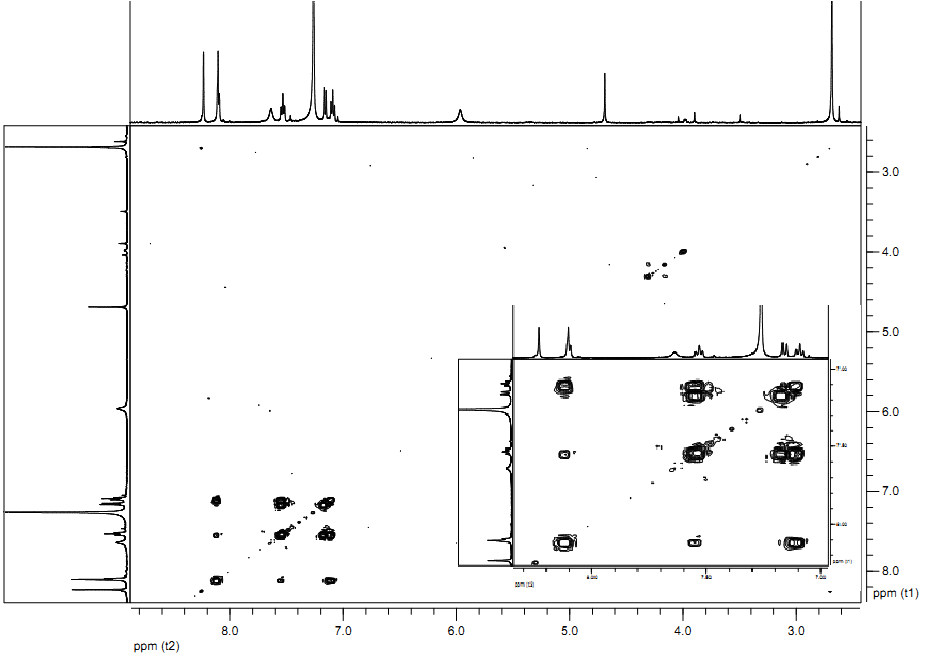


(**F**)


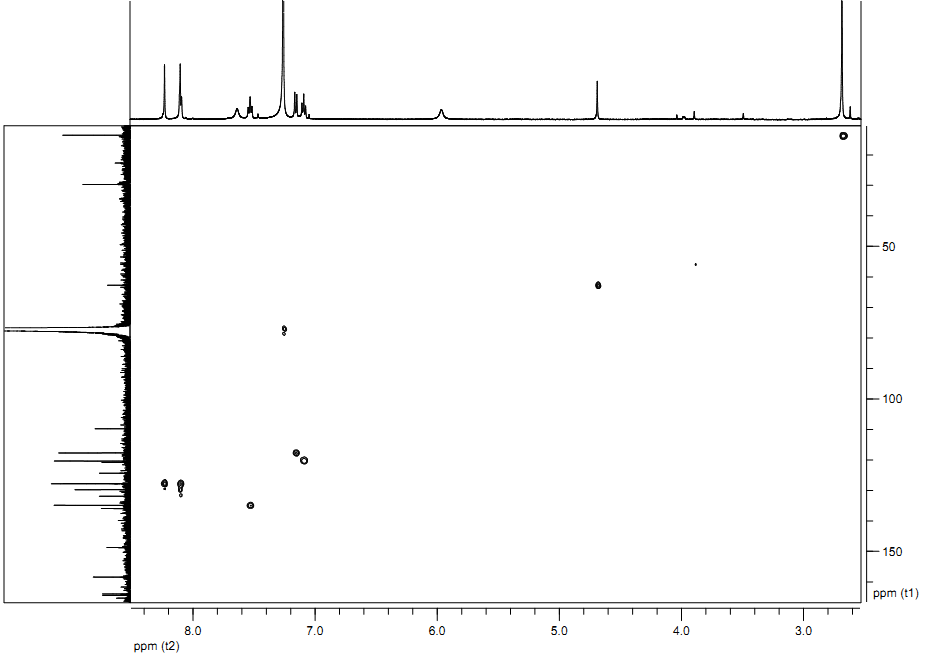


(**G**)

**Figure S5.** *Cont.*


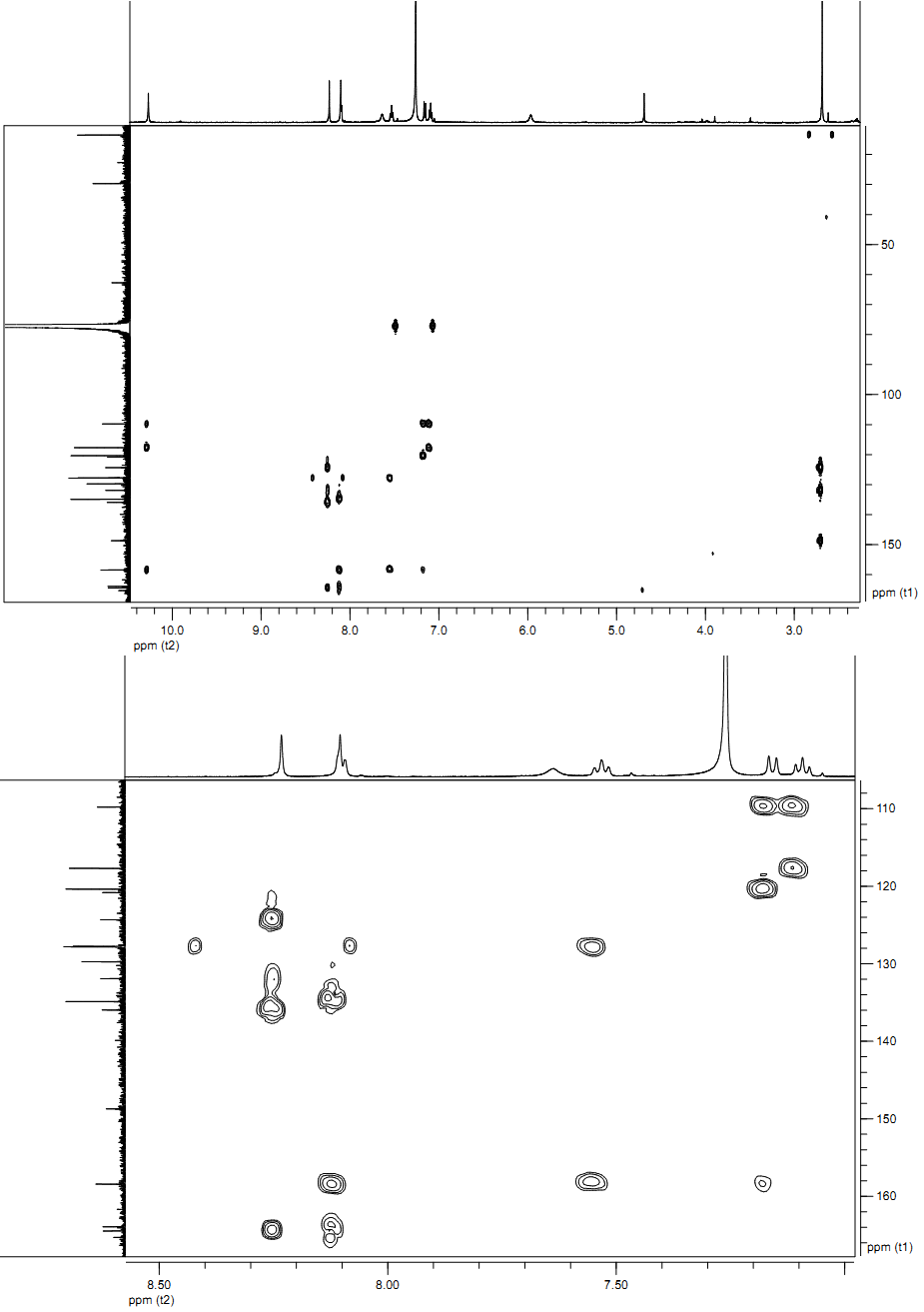


(**H**)

**Figure S5.** (**A**) HR-ESI-MS spectrum of **4**; (**B**) UV spectrum of **4**; (**C**) ^1^H-NMR spectrum (500 MHz, *CDCl_3_*) of **4**; (**D**) ^13^C-NMR spectrum (125 MHz, *CDCl_3_*) of **4**; (**E**) DEPT 135 spectrum (125 MHz, *CDCl_3_*) of **4**; (**F**) ^1^H–^1^H-COSY spectrum (500 × 500 MHz, *CDCl_3_*) of **4**; (**G**) ^1^H–^13^C-HSQC spectrum (500 × 125 MHz, *CDCl_3_*) of **4**; (**H**) ^1^H–^13^C-HMBC spectrum (500 × 125 MHz, *CDCl_3_*) of **4**.

(**A**)

(**B**)

**Figure S6.** *Cont.*

(**C**)

**Figure S6. (A)** HR-ESI-MS spectrum of **5**; **(B)** UV spectrum of **5**; **(C)** HR-ESI-MS/MS comparison of **3** and **5**.


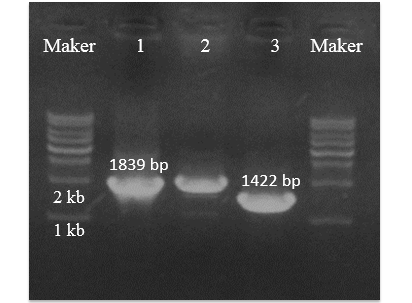


**Figure S7.** PCR analysis of Δ*1741* double-crossover mutant. Lane 1, pKC1139::*1741*::*neo*; lane 2, Δ*1741* mutant strain; lane 3, wild-type strain *S.* *olivaceus* FXJ8.012.

**Table S1.** Strains and plasmids used in this study.

| **Strain/plasmid** | **Description** | **Reference or source** |
| --- | --- | --- |
| Strains |  |  |
| *E. coli* Top 10 | For plasmid isolation and confirmation | Invitrogen |
| *E. coli* ET12567/pUZ8002 | For intergeneric conjugation | [1,2] |
| *Streptomyce galbus* FXJ1.235 | Producer of mycemycins A and B | This study |
| *Streptomyces olivaceus* FXJ8.012 | Wild-type strain | This study |
| *Streptomyces olivaceus* FXJ8.012Δ*1741* | Producer of mycemycins C, D and E | This study |
| Plasmids |  |  |
| pKC1139 | *E. coli*-*Streptomyces* shuttle vector | [3] |
| pUZ8002 | Facilitates transfer of genetic material by conjugation | [4] |
| pKC1139::*1741*::*neo* | Orf-*1741* deletion | This study |
| pUC119::*neo* | Source of *neo* (kana^R^) | [5] |

**Table S2.** Primers used in this study (restriction sites used are underlined; protective nucleotides are in italics).

| **Primer Name** | **Primer Sequence (5′**–**3′)** |
| --- | --- |
| *1741*-L-Forward | *CCC*AAGCTTCGTGGATACGGGAGACGAGGAAGAC |
| *1741*-L-Reverse | *GC*TCTAGAGAATTCGTCAGTCGCCTTTGTGTCCGTCG |
| *1741*-R-Forward | *G*GAATTCGGTAGCCGAAAGTCCGGTGCTT |
| *1741*-R-Reverse | *GC*TCTAGAAAGTGGTAGGCGAGTGCGAGGTC |
| Δ*1741*-Foward | GACACTCACGCATCACGGTCTGGC |
| Δ*1741*-Reverse | CTGAGCACTCGGTTGGGACTGGC |
| *neo*-*Eco*RI-Forward | *G*GAATTCATCCCCTGGATACCGCTCGCCGCAG |
| *neo*-*Eco*RI-Reverse | *G*GAATTCTACCCGAACCCCAGAGTCCCG |

References

1. Gust, B.; Chandra, G.; Jakimowicz, D.; Yuqing, T.; Bruton, C.J.; Chater, K.F. Lambda red-mediated genetic manipulation of antibiotic-producing *Streptomyces*. *Adv. Appl. Microbiol.* **2004**, *54*, 107–128.
2. Gust, B.; Challis, G.L.; Fowler, K.; Kieser, T.; Chater, K.F. PCR-targeted *Streptomyces* gene replacement identifies a protein domain needed for biosynthesis of the sesquiterpene soil odor geosmin. *Proc. Natl. Acad. Sci.* **2003**, *100*, 1541–1546.
3. Kieser, T.; Bibb, M.J.; Buttner, M.J.; Chater, K.F.; Hopwood, D.A. *Practical Streptomyces Genetics*; John Innes Foundation: Colney, Norwich, England, 2000.
4. Bierman, M.; Logan, R.; O’Brien, K.; Seno, E.T.; Rao, R.N.; Schoner, B.E. Plasmid cloning vectors for the conjugal transfer of DNA from Escherichia coli to *Streptomyces* spp. *Gene* **1992**, *116*, 43–49.
5. Pan, Y.Y.; Liu, G.; Yang, H.Y.; Tian, Y.Q.; Tan, H.R. The pleiotropic regulator AdpA-L directly controls the pathway-specific activator of nikkomycin biosynthesis in *Streptomyces ansochromogenes*. *Mol. Microbiol.* **2009**, *72*, 710–723.

© 2015 by the authors; licensee MDPI, Basel, Switzerland. This article is an open access article distributed under the terms and conditions of the Creative Commons Attribution license (http://creativecommons.org/licenses/by/4.0/).
